# Transition Semantics for Branching Time

**DOI:** 10.1007/s10849-015-9231-6

**Published:** 2015-11-06

**Authors:** Antje Rumberg

**Affiliations:** Department of Philosophy, University of Konstanz, Postfach 17, 78457 Konstanz, Germany

**Keywords:** Branching time, Transitions, Stability, Future contingents

## Abstract

In this paper we develop a novel propositional semantics based on the framework of branching time. The basic idea is to replace the moment-history pairs employed as parameters of truth in the standard Ockhamist semantics by pairs consisting of a moment and a consistent, downward closed set of so-called *transitions*. Whereas histories represent complete possible courses of events, sets of transitions can represent incomplete parts thereof as well. Each transition captures one of the alternative immediate future possibilities open at a branching point. The transition semantics exploits the structural resources a branching time structure has to offer and provides a fine-grained picture of the interrelation of modality and time. In addition to temporal and modal operators, a so-called *stability* operator becomes interpretable as a universal quantifier over the possible future extensions of a given transition set. The stability operator allows us to specify how and how far time has to unfold for the truth value of a sentence at a moment to become settled and enables a perspicuous treatment of future contingents. We show that the semantics developed along those lines generalizes and extends extant approaches: both Peirceanism and Ockhamism can be viewed as limiting cases of the transition approach that build on restricted resources only, and on both accounts, stability collapses into truth.

## Introduction

The interaction of modality and time became a topic of formal investigation early on in the development of tense logic by Prior (1957). The initial suggestion was to understand modality in terms of quantification over moments in a linear temporal structure—the so-called Diodorean modality. Kripke suggested a branching representation of the interaction of modality and time instead.^1^ Formal languages based on branching time structures were studied in Prior (1967), and Thomason (1970) contains the first detailed overview of a logic based on those structures. Branching time structures allow for a direct representation of alternative future possibilities, all of which share some common past. Formally, a *branching time structure* is defined as a non-empty strict partial ordering of moments that is connected and left-linear. Each maximal linear set of moments in such an ordering represents a *history*, a complete possible course of events.

The crucial point in developing a propositional semantics on a branching time structure consists in specifying appropriate truth conditions for the future operator. At a given moment, there may be more than one possibility for the future, with nothing yet deciding between them.^2^ For concreteness assume that you have purchased a ticket for tomorrow’s lottery. Given that the outcome of the drawing is in fact objectively indeterminate, it is both possible that your ticket will win and that your ticket will lose, with nothing yet to tip the balance. Whether your ticket will win or lose, depends on how the future unfolds, or more precisely, on the outcome of tomorrow’s drawing.

There are two popular semantic approaches based on the framework of branching time. Prior (1967) refers to them as *Peirceanism* and *Ockhamism*. On the Peircean account, the semantic evaluation is relativized to a moment parameter only, and the future operator requires a witness in every possible future continuation of the moment of evaluation. Truth coincides with inevitability. In our lottery example, the Peircean semantics renders both the sentence “Your ticket will win” and the sentence “Your ticket will lose” false at any moment preceding the drawing.

The Ockhamist account, on the other hand, makes use of a history as a second parameter of truth next to the moment parameter and thereby generalizes Peirceanism. Modal operators become interpretable as quantifiers over histories, and truth and inevitability come apart. In the case of the future operator, the moment of evaluation is simply shifted forward on the given history, just as in linear tense logic. In our example, at any moment before the drawing, the sentence “Your ticket will win” is true with respect to one history, while the contrary prediction “Your ticket will lose” is true with respect to another history, rendering both outcomes possible.

The semantics we are proposing generalizes and extends both Peirceanism and Ockhamism. The basic idea is to replace the moment-history pairs employed as parameters of truth in the Ockhamist semantics by pairs consisting of a moment and a consistent, downward closed set of so-called *transitions*. Each transition captures one of the alternative immediate future possibilities open at a branching point. Whereas histories represent complete possible courses of events, sets of transitions can represent incomplete parts thereof as well. Our future operator has both Peircean and Ockhamist traits: it demands a future witness in every possible future continuation of the moment of evaluation that is an extension of the given transition set. In addition to temporal and modal operators, we introduce a so-called *stability* operator into our language, which is interpreted as a universal quantifier over the possible future extensions of a given transition set. With that operator at our disposal, we cannot only express that, at any moment before the drawing, it is both possible that your ticket will win and that your ticket will lose, but also specify how and how far time has to unfold for things to become settled one way or the other. Truth and stability come apart. At any moment before the drawing, the sentences “Your ticket will win” and “Your ticket will lose” are neither stably-true nor stably-false but contingent with respect to the past course of events up to that moment. Their truth values only stabilize as the future unfolds. It is the drawing that constitutes the relevant tipping point: with respect to any incomplete course of events that encompasses one of the possible outcomes of tomorrow’s drawing, each of our sentences is either stably-true or stably-false at any moment before the drawing. Any incomplete course of events that captures one of the possible outcomes of the drawing suffices to settle matter, even though there might be several histories that contain the very same outcome of the drawing and differ only with respect to what will happen hundreds or thousands of years hence.

By exploiting the structural resources a branching time structure has to offer, the transition semantics allows for a fine-grained picture of the interrelation of modality and time. With its stability operator, it enables a perspicuous treatment of future contingents and gains expressive means that are not available on extant accounts. We show that the semantics developed along those lines generalizes and extends both Peirceanism and Ockhamism: both accounts can be viewed as limiting cases of the transition approach, and Peircean and Ockhamist validity are definable in the transition semantics. Both Peirceanism and Ockhamism are obtained by restricting the range of transition sets that are taken into account in the semantic evaluation, and on both accounts, stability collapses into truth.

The paper is structured as follows: in Sect. 2 we introduce the framework of branching time and briefly discuss the Peircean and the Ockhamist account. In Sect. 3 we define the notion of a transition and that of a consistent, downward closed transition set. We then provide the recursive semantic clauses of the transition semantics we are proposing and illustrate how sentences about the future are treated within that semantic framework. In Sect. 4 we show that the transition approach generalizes both Peirceanism and Ockhamism and illustrate how it extends extant accounts by its notion of stability.

## Branching Time

In this section we introduce the notion of a branching time structure (Sect. 2.1), provide a general definition of a branching time model and briefly discuss the Peircean and the Ockhamist account (Sect. 2.2).

### Branching Time Structures

In the Prior-Thomason theory of branching time, the modal-temporal structure of the world is represented as a tree of histories that share some common past and branch toward the future. We define a *branching time structure* as a non-empty strict partial ordering of moments, $$\mathcal {M}=\langle M,<\rangle $$, such that (BT1) the temporal ordering $$<$$ on *M* is left-linear, (BT2) any two moments in *M* have a greatest common lower bound in *M*, and (BT3) *M* has no maximal elements. Condition (BT1) captures the idea that the past is fixed while the future is open. It requires the partial ordering to be tree-like: there is no backward branching. Condition (BT2) first of all secures the unity of the structure by demanding the temporal ordering $$<$$ to be connected: any two moments have a common lower bound. The requirement that there be a *greatest* common lower bound in each case is inserted here as it allows us to define branching points, which figure as the initials of our transitions, in a perspicuous way. Condition (BT3) expresses the idea that time does not end, i.e., there is no last moment. As usual, we use $$m\le m'$$ to stand for $$(m<m'$$ or $$m=m')$$.

#### **Definition 1**

(*BT structure*) A *BT structure*$\mathcal {M}=\langle M,<\rangle$ is a non-empty strict partial order (i.e. a set $M \ne \emptyset$ together with a relation $<$ that is irreflexive, asymmetric and transitive) such that

(BT1): $$\text {for all } m,m',m'' \in M\text {, if } m'<m \text { and } m''<m \text {, then } m'\le m'' \text { or } m''\le m'$$;
(BT2): $$\text {for all } m,m' \in M \text {, there is some } m''\in M \text { s.t. } m''\le m \text {, } m'' \le m' \text { and for all} m''' \in M \text {, if } m'''\le m \text { and }m'''\le m' \text {, then } m'''\le m''$$;
(BT3): $$\text {for all } m \in M \text {, there is some } m'\in M \text { s.t. } m<m'$$.

We do not place any further restrictions on the earlier-later relation $<$ that represents the temporal ordering of moments. We allow it to be discrete, dense or continuous. While discrete structures are important for applications in computer science, in a more general setting triggered by philosophical considerations, dense and continuous structures should be allowed for as well.

A set of moments is modally consistent if it is possible that those moments co-occur within a single possible course of events, viz. if the set is linearly ordered via $<$. For $\mathcal {M}=\langle M,<\rangle$ a BT structure, a *history* is a maximal linear subset of *M*. As a maximal $<$-chain in *M*, a history is a maximal modally consistent set of moments and represents a complete possible course of events.

#### **Definition 2**

(*History*) Given a BT structure $$\mathcal {M}=\langle M,<\rangle $$, a set $$h \subseteq M$$ is a *history* iff *h* is a maximal linear subset in *M*, i.e. a subset that is linearly ordered via $$<$$$$(\text {for all } m,m'\in h\text {, } m\le m' \text { or } m'\le m)$$ and such that there is no proper superset $$h' \supsetneq h$$ in *M* that is linearly ordered via $$<$$ as well. The *set of histories in*$$\mathcal {M}$$  is denoted by $$\mathsf {hist}(\mathcal {M})$$. For a moment $$m \in M$$, the *set of histories containingm* is denoted by $${\mathsf {H}}_m$$; so $${\mathsf {H}}_m = \{h \in \mathsf {hist}(\mathcal {M})\mid m \in h\}$$.

Due to the absence of backward branching (BT1), histories are *downward closed*: a history *h* contains for any moment $m \in h$, all moments $m'$ in that moment’s past, i.e., if $m \in h$ and $m'<m$, then $m' \in h$. In particular, we thus have that if $m'\le m$, then ${\mathsf {H}}_m \subseteq {\mathsf {H}}_{m'}$. The fact that histories are downward closed implies together with condition (BT2) that the intersection of any two histories is non-empty and contains a greatest element. This greatest element constitutes a *branching point*. Condition (BT1) and (BT2) of Definition 1 thus jointly assure that any two histories branch at some moment.

### Branching Time Semantics

BT structures can be employed in the semantics of formal languages containing temporal and modal operators—in fact, the branching time framework was developed for those semantic purposes in the context of Prior’s tense logic. The languages we consider throughout this paper—the Peircean, the Ockhamist and the transition language—each extend the standard propositional language ${\mathscr {L}}$ (with propositional variables and the usual Boolean connectives) by a certain set of temporal and/or modal operators. The semantics of those three languages differ with respect to which structural elements—over and above a moment of evaluation—are employed as parameters of truth in the recursive semantic machinery. We spell out the definition of a branching time model and the recursive semantic clauses for the truth-functional connectives in full generality, using “$\mathcal {I}$ ” to stand for the respective set of indices of evaluation and “$\imath$” for one such index in $\mathcal {I}$, which includes at least a moment parameter. We denote the set of propositional variables of the basic propositional language ${\mathscr {L}}$ by ${{\mathsf {A}}}{{\mathsf {t}}}$ and make use of negation $\lnot$ and conjunction $\wedge$ as the only primitive truth-functional connectives.

A *BT model* is defined as a pair ${\mathfrak {M}}=\langle \mathcal {M},v\rangle$, where $\mathcal {M}=\langle M,<\rangle$ is a BT structure and *v* a valuation function that assigns truth values to the propositional variables $p \in {{\mathsf {A}}}{{\mathsf {t}}}$ relative to an index of evaluation $\imath \in \mathcal {I}$.^3^

#### **Definition 3**

(*BT model*) A *BT model*${\mathfrak {M}}=\langle \mathcal {M},v\rangle$ is a BT structure $\mathcal {M}=\langle M,<\rangle$ together with a valuation function $v: {{\mathsf {A}}}{{\mathsf {t}}}\times \mathcal {I}\rightarrow \{0,1\}$.

The truth of a sentence $\phi \in {\mathscr {L}}$ in a model ${\mathfrak {M}}=\langle \mathcal {M},v\rangle$ at an index of evaluation $\imath \in \mathcal {I}$, in symbols: ${\mathfrak {M}},\imath \vDash \phi$, is now defined recursively:

($${{\mathsf {A}}}{{\mathsf {t}}}$$)
$${\mathfrak {M}},\imath \vDash p \text { iff } v(p,\imath ) = 1$$;
($$\lnot $$)
$${\mathfrak {M}},\imath \vDash \lnot \phi \text { iff } {\mathfrak {M}},\imath \not \vDash \phi $$;
($$\wedge $$)
$${\mathfrak {M}},\imath \vDash \phi \wedge \psi \text { iff } {\mathfrak {M}},\imath \vDash \phi \text { and } {\mathfrak {M}},\imath \vDash \psi $$.

The semantic clauses for the particular temporal and/or modal operators, that extend the valuation on the sentences of ${\mathscr {L}}$ to all sentences of the respective language, are provided as those languages are specified in detail.

As mentioned above, the most popular semantic approaches based on the framework of branching time are what Prior (1967) calls the *Peircean* and the *Ockhamist* approach, and we now briefly discuss them in turn.^4^

#### Peirceanism

The Peircean language ${\mathscr {L}}_{{\mathsf {p}}}$ extends the propositional language ${\mathscr {L}}$ by a future operator $\mathsf {F_p}$ and a past operator $\mathsf {P_p}$. On the Peircean account, the recursive semantic machinery is relativized to a single parameter of truth: the semantic evaluation on a BT structure $\mathcal {M}=\langle M,<\rangle$ depends solely on a moment of evaluation $m \in M$. The semantic clause for the future operator $\mathsf {F_p}$ universally quantifies over all histories containing the moment of evaluation, demanding a future witness in every single one of them. That is, a sentence of the form $\mathsf {F_p}\phi$ is true at a moment $m \in M$ iff every history passing through *m* contains some later moment $m'>m$ at which $\phi$ is true. The semantic clause for the past operator $\mathsf {P_p}$ is straightforward: a sentence of the form $\mathsf {P_p}\phi$ is true at a moment $m \in M$ iff $\phi$ is true at some earlier moment $m'<m$. Since every moment has a unique past, no universal quantification is needed in that case.

Below the semantic clauses for the future operator $\mathsf {F_p}$ and past operator $\mathsf {P_p}$ are provided. We use ${\mathfrak {M}},m \vDash _{{\mathsf {p}}}\phi$ in order to indicate that a sentence $\phi \in {\mathscr {L}}_{{\mathsf {p}}}$ is true at a moment *m* in a Peircean model ${\mathfrak {M}}= \langle \mathcal {M},v_{\mathsf {p}}\rangle$ on a BT structure $\mathcal {M}=\langle M,<\rangle$ with $v_{\mathsf {p}}: {{\mathsf {A}}}{{\mathsf {t}}}\times M \rightarrow \{0,1\}$ according to the Peircean semantics.

($$\mathsf {F_p}$$)
$${\mathfrak {M}},m \vDash _{{\mathsf {p}}}\mathsf {F_p}\phi \text { iff for all } h \in {\mathsf {H}}_m \text {, there is some } m' \in h \text { s.t. } m'>m \,\text { and } {\mathfrak {M}}, m' \vDash _{{\mathsf {p}}} \phi $$;
($$\mathsf {P_p}$$)
$${\mathfrak {M}},m \vDash _{{\mathsf {p}}}\mathsf {P_p}\phi \text { iff there is some } m'<m \text { s.t. } {\mathfrak {M}},m' \vDash _{{\mathsf {p}}}\phi $$.

#### Ockhamism

The Ockhamist language ${\mathscr {L}}_{{\mathsf {o}}}$ enriches the propositional language ${\mathscr {L}}$ by a future operator $\mathsf {F_o}$ and a past operator $\mathsf {P_o}$, as well as by an operator for inevitability, or settledness, $\mathsf {\Box _o}$. On the Ockhamist account, the semantic evaluation on a BT structure $\mathcal {M}=\langle M,<\rangle$ depends on a history as a second parameter of truth next to the moment parameter. That is, truth at a moment of evaluation is relativized to a complete possible course of events. Sentences are evaluated at moment-history pairs *m* / *h*, where $m \in M$ and $h \in {\mathsf {H}}_m$. In the case of the temporal operators $\mathsf {F_o}$ and $\mathsf {P_o}$, the moment of evaluation is shifted forward or backward, respectively, on the given history, just as in linear tense logic. A sentence of the form $\mathsf {F_o}\phi$ is true at a moment-history pair *m* / *h* iff the history *h* contains a later moment $m'>m$ at which $\phi$ is true with respect to *h*. The past operator is interpreted analogously. Note that in the case of the past operator the requirement $m' \in h$ can be dropped since histories are downward closed. We use ${\mathfrak {M}},m/h \vDash _{{\mathsf {o}}}\phi$ in order to indicate that a sentence $\phi \in {\mathscr {L}}_{{\mathsf {o}}}$ is true at a moment-history pair *m* / *h* in an Ockhamist model ${\mathfrak {M}}= \langle \mathcal {M},v_{\mathsf {o}}\rangle$ on a BT structure $\mathcal {M}=\langle M,<\rangle$ with $v_{\mathsf {o}}: {{\mathsf {A}}}{{\mathsf {t}}}\times \{m/h: m\in M \text { and } h \in {\mathsf {H}}_m\} \rightarrow \{0,1\}$ according to the Ockhamist semantics.

($$\mathsf {F_o}$$)
$${\mathfrak {M}},m/h \vDash _{{\mathsf {o}}}\mathsf {F_o}\phi \text { iff there is some } m'\in h \text { s.t. } m'>m \text { and } {\mathfrak {M}},m'/h \vDash _{{\mathsf {o}}}\phi $$;
($$\mathsf {P_o}$$)
$${\mathfrak {M}},m/h \vDash _{{\mathsf {o}}}\mathsf {P_o}\phi \text { iff there is some } m'<m \text { s.t. } {\mathfrak {M}},m'/h \vDash _{{\mathsf {o}}}\phi $$.

Since the semantic evaluation is relativized to a history as a second parameter of truth, modal operators become interpretable as quantifiers over the set of histories containing the moment of evaluation. The inevitability operator $\mathsf {\Box _o}$ amounts to universal quantification. A modal operator for possibility, $\mathsf {\Diamond _o}$, can be defined as its dual, i.e., $\mathsf {\Diamond _o}:= \lnot \mathsf {\Box _o}\lnot$. The modality involved is ${{\mathsf {S}}}{{\mathsf {5}}}$.

($$\mathsf {\Box _o}$$)
$${\mathfrak {M}},m/h \vDash _{{\mathsf {o}}}\mathsf {\Box _o}\phi \text { iff for all } h'\in {\mathsf {H}}_m, {\mathfrak {M}},m/h' \vDash _{{\mathsf {o}}}\phi $$.

By making use of a history as a second parameter of truth next to the moment parameter, Ockhamism generalizes Peirceanism. Modal operators become interpretable, and truth and inevitability come apart while they coincide on the Peircean account. On the Ockhamist account, a sentence can be assigned different truth values at the very same moment with respect to different histories. The Peircean future operator $\mathsf {F_p}$ is equivalent to the composition $\mathsf {\Box _o}\mathsf {F_o}$ of Ockhamist operators. Due to the dependence of the semantic evaluation at a moment on a history parameter, Ockhamism gains expressive means that are not available on the Peircean account.

## Transition Semantics

As said, by relativizing the semantic evaluation at a moment to a history parameter, that specifies some possible course of events, Ockhamism generalizes Peirceanism. Histories, however, represent *complete* possible courses of events, whereas in general an *incomplete* possible course of events suffices for settling the truth or the falsity of a sentence. In the lottery example that we considered in the introduction, it is the drawing that constitutes the relevant tipping point: with respect to any incomplete course of events that encompasses one of the possible outcomes of the drawing it is settled at any moment before the drawing whether your ticket will win or lose. By replacing the history parameter employed in the Ockhamist semantics by a consistent, downward closed set of transitions, we allow the semantic evaluation to be relativized to incomplete possible courses of events as well. That sets of transitions can serve as a local alternative to histories was suggested in Müller (2014). The transition semantics we are proposing here goes significantly beyond that suggestion: by enriching the purely temporal language considered in Müller (2014) by modal operators and a stability operator, we obtain a language that is able to reflect the richness of the semantics. The stability operator allows us to specify how and how far time has to unfold for the truth value of a sentence at a moment to become settled and enables a perspicuous treatment of future contingents. The semantics developed along those lines provides a fine-grained picture of the interrelation of modality and time and generalizes and extends both Peirceanism and Ockhamism.

In this section we first define the notion of a transition and that of a consistent, downward closed transition set (Sect. 3.1). We then put forth the recursive semantic clauses of the transition semantics we are proposing (Sect. 3.2) and illustrate how sentences about the future are treated within that semantic framework (Sect. 3.3).

### Transitions

Transitions provide a local alternative to histories. Whereas a history represents a complete possible course of events, a transition captures one of the alternative immediate future possibilities open at a branching point. When introducing the Prior-Thomason theory of branching time in Sect. 2.1 above, we said that the conditions (BT1) and (BT2) in the definition of a branching time structure (Definition 1) jointly assure that the intersection of any two histories is non-empty and contains a greatest element. This greatest element constitutes a *branching point*. The definition of a BT structure thus guarantees that any two histories branch at some moment. If the moment *m* is a branching point, not all pairs of histories containing *m* need to branch at *m*, however; some might still continue to overlap for a certain while after *m*, in which case they are said to be *undivided-at*-*m*. At each branching point, some pairs of histories branch, others are undivided. For two histories to be undivided at a moment *m*, it is both necessary and sufficient that they share some moment $m'$ later than *m*, which by downward closedness of histories implies that they also share *m*.

#### **Definition 4**

(*Undividedness-at-mand branching-at-m*) Given a BT structure $\mathcal {M}=\langle M,<\rangle$ and a moment $m \in M$, two histories $h,h' \!\in \! \mathsf {hist}(\mathcal {M})$ are *undivided-at-m*, in symbols: $h \equiv _m h'$, iff there is some $m' \in M$ such that $m' > m$ and $m' \in h \cap h'$. Two histories $h,h' \in \mathsf {hist}(\mathcal {M})$*branch-at-m*, in symbols: $h \perp _m h'$, iff the moment *m* is the greatest element in $h \cap h'$ (i.e., $m \in h \cap h'$ and for all $m' \in h \cap h'$, $m'\le m$). If for a moment *m*, there are histories $h,h' \in \mathsf {hist}(\mathcal {M})$ s.t. $h \perp _m h'$, we call *m* a *branching point*.

Definition 4 implies that two histories that share some moment *m* are undivided-at-*m* if and only if they do not branch-at-*m*. The relation of undividedness-at-*m* is an equivalence relation on the set ${\mathsf {H}}_m$ of histories containing the moment *m* and thus yields a partition ${\varPi }_m$ of that set. The partition ${\varPi }_m$ of ${\mathsf {H}}_m$ is non-trivial, i.e., ${\varPi }_m \ne \{{\mathsf {H}}_m\}$, if and only if the moment *m* is a branching point. The equivalence class of a history $h \in {\mathsf {H}}_m$ with respect to the relation of undividedness-at-*m* is denoted by $[h]_m$, so $[h]_m = \{h' \in {\mathsf {H}}_m\mid h\equiv _m h'\}$. From the definition of undividedness it immediately follows that all histories that share some moment *m* are undivided at any moment $m'$ in that moment’s past: if $h,h' \in {\mathsf {H}}_m$ and $m'<m$, then $h \equiv _{m'} h'$, and hence ${\mathsf {H}}_m \subseteq [h]_{m'} = [h']_{m'}$. In particular, it holds that any two histories that are undivided at some moment *m* are also undivided at any earlier moment $m'$: if $h \equiv _m h'$ and $m'<m$, then $h \equiv _{m'} h'$, and consequently $[h]_m = [h']_m \subseteq [h]_{m'} = [h']_{m'}$.

The notion of a branching point and the relation of undividedness play a crucial role in the definition of a transition. At each branching point *m*, the set ${\mathsf {H}}_m$ of histories containing *m* is partitioned into sets of histories that are undivided-at-*m*, and each such set specifies one of the possible immediate future continuations of *m*. A transition links a branching point *m* to one such local future possibility $H \in {\varPi }_m$. It picks out one of the alternative immediate future possibilities open at that moment. We define a *transition* as a pair $\langle m,H\rangle$, also written $\langle m\!\rightarrowtail \!H\rangle$, consisting of a branching point $m \in M$ and a set of locally undivided histories $H \in {\varPi }_m$.^5^ We call the moment $m \in M$ the *initial* of the transition $\langle m\!\rightarrowtail \!H\rangle$ and the set of histories $H \in {\varPi }_m$ its *outcome*. For every branching point *m*, there are at least two transitions that share the initial *m* but have pairwise disjoint outcomes.

#### **Definition 5**

(*Transition*) For $\mathcal {M}=\langle M,<\rangle$ a BT structure, a *transition* is a pair $\langle m,H\rangle$, also written $\langle m\!\rightarrowtail \!H\rangle$, with $m \in M$ a branching point and $H \in {\varPi }_m$. The moment *m* is the *initial* and the set of histories *H* the *outcome* of the transition $\langle m\!\rightarrowtail \!H\rangle$. The *set of transitions in*$\mathcal {M}$ is denoted by $\mathsf {trans}(\mathcal {M})$.^6^

The complexity of the definition of a transition is due to the fact that the definition is intended to be general enough to accommodate the case of dense and continuous BT structures. In case $\mathcal {M}=\langle M,<\rangle$ is discrete, so that for every branching point $m \in M$, there is a non-singleton set $\mathsf {succ}(m)$ of immediate successors of *m*, a transition can be defined as a pair of moments $\langle m,m'\rangle$ with $m' \in \mathsf {succ}(m)$. This is the kind of notion of a transition that is prevalent in computer science, where primarily discrete structure are studied. If the BT structure under consideration is not discrete, however, transitions cannot generally be defined as pairs of moments. In order to see this, consider a BT structure that contains a history *h* isomorphic to the real numbers ${\mathbb {R}}$ such that at any moment $m_i \in h$ with $i \in {\mathbb {R}}$, the partition ${\varPi }_{m_i}$ is non-trivial, i.e., ${\varPi }_{m_i} \ne \{{\mathsf {H}}_{m_i}\}$. For every moment $m_i \in h$, we have a transition $\langle m_i\!\rightarrowtail \!H\rangle$ with $h \in H$ that is irreducible to a pair of moments.

Rather than focusing on single transitions, we consider whole sets of transitions, which allows for even more generality. A set of transitions can be said to be modally consistent if there is at least one possible course of events that is admitted by all the transitions in the set. In other words, a consistent set of transitions is such that it allows at least one history to occur but may exclude others. The *set of histories allowed by a set of transitionsT*, ${\mathsf {H}}(T)$, is the intersection of the outcomes of those transitions.

#### **Definition 6**

(*The set of histories allowed byT*) Given a BT structure $\mathcal {M}=\langle M,<\rangle$ and a transition set $T \subseteq \mathsf {trans}(\mathcal {M})$, the *set of histories allowed byT*, in symbols: ${\mathsf {H}}(T)$, is given by ${\mathsf {H}}(T)= \bigcap _{\langle m \rightarrowtail H\rangle \in T} H$.

We can now define a set of transitions *T* to be *consistent* iff the set ${\mathsf {H}}(T)$ of histories allowed by *T* is non-empty.

#### **Definition 7**

(*Consistency*) For $\mathcal {M}=\langle M,<\rangle$ a BT structure, a transition set $T \subseteq \mathsf {trans}(\mathcal {M})$ is *consistent* iff ${\mathsf {H}}(T)\ne \emptyset$.

Consistency of a transition set comes down to the requirement that all transitions in the set have to lie within one chain. A consistent set of transitions can in particular not contain two different transitions with the same initial. Given a BT structure $\mathcal {M}=\langle M,<\rangle$, we can define a strict partial ordering on the set of transitions $\mathsf {trans}(\mathcal {M})$ in terms of an inclusion relation on the outcomes. A transition $\langle m\!\rightarrowtail \!H\rangle \in \mathsf {trans}(\mathcal {M})$*precedes* a transition $\langle m'\!\rightarrowtail \!H'\rangle \in \mathsf {trans}(\mathcal {M})$ iff the outcome of $\langle m'\!\rightarrowtail \!H'\rangle$ is properly included in the outcome of $\langle m\!\rightarrowtail \!H\rangle$.

#### **Definition 8**

(*Transition ordering*) Given a BT structure $\mathcal {M}=\langle M,<\rangle$ and transitions $\langle m\!\rightarrowtail \!H\rangle , \langle m'\!\rightarrowtail \!H'\rangle \in \mathsf {trans}(\mathcal {M})$, we say that $\langle m\!\rightarrowtail \!H\rangle$*precedes*$\langle m'\!\rightarrowtail \!H'\rangle$, in symbols: $\langle m\!\rightarrowtail \!H\rangle \prec \langle m'\!\rightarrowtail \!H'\rangle$, iff $H' \subsetneq H$.

Note that for $\langle m\!\rightarrowtail \!H\rangle , \langle m'\!\rightarrowtail \!H'\rangle \in \mathsf {trans}(\mathcal {M})$, we have $H' \subsetneq H$ if and only if both $m<m'$ and $H \cap H' \ne \emptyset$. Just as the temporal earlier-later relation $<$ on *M*, the transition ordering $\prec$ on $\mathsf {trans}(\mathcal {M})$ is left-linear. We can prove that a set of transitions $T \subseteq \mathsf {trans}(\mathcal {M})$ is consistent if and only if all transitions in the set are linearly ordered via $\prec$.

#### **Proposition 1**

For $\mathcal {M}=\langle M,<\rangle$ a BT structure and $T \subseteq \mathsf {trans}(\mathcal {M})$ a set of transitions, we have that

$$\begin{aligned} {\mathsf {H}}(T)\ne \emptyset \quad \text { iff } \quad T\text { is linearly ordered via }\prec . \end{aligned}$$

#### *Proof*

“$\Rightarrow$”: Assume that ${\mathsf {H}}(T)\ne \emptyset$. Then there is some history $h \in {\mathsf {H}}(T)$, and we have that $\{m \in M\mid \langle m\!\rightarrowtail \!H\rangle \in T\} \subseteq h$. By Definition 2 it follows that $\{m \in M\mid \langle m\!\rightarrowtail \!H\rangle \in T\}$ is linearly ordered via the relation $<$. Let $\langle m'\!\rightarrowtail \!H'\rangle$, $\langle m''\!\rightarrowtail \!H''\rangle \in T$. Then $m',m'' \in \{m \in M\mid \langle m\!\rightarrowtail \!H\rangle \in T\}$. Three cases can be considered: (i) if $m'=m''$, then $H'=H''$, since otherwise ${\mathsf {H}}(T)\ne \emptyset$; (ii) if $m'<m''$, then $H'' = [h]_{m''} \subsetneq [h]_{m'} = H'$, which implies $\langle m'\!\rightarrowtail \!H'\rangle \prec \langle m''\!\rightarrowtail \!H''\rangle$; (iii) if $m''<m'$, then $H' = [h]_{m'} \subsetneq [h]_{m''} = H''$ and thus $\langle m''\!\rightarrowtail \!H''\rangle \prec \langle m'\!\rightarrowtail \!H'\rangle$.

“$\Leftarrow$”: Assume that *T* is linearly ordered via $\prec$. If *T* contains a maximal element $\langle m\!\rightarrowtail \!H\rangle$, then by Definition 8 we have that $H \subseteq {\mathsf {H}}(T)$ and thus ${\mathsf {H}}(T)\ne \emptyset$. Assume that *T* does not contain a maximal element. Since *T* is linearly ordered via $\prec$, it follows by Definition 8 that $\{H \subseteq \mathsf {hist}(\mathcal {M})\mid \langle m\!\rightarrowtail \!H\rangle \in T\}$ is linearly ordered via proper set inclusion $\subsetneq$, which in turn implies that the set $\{m \in M\mid \langle m\!\rightarrowtail \!H\rangle \in T\}$ is linearly ordered via $<$. Hence, by the Axiom of Choice, there is some history $h \supseteq \{m \in M\mid \langle m\!\rightarrowtail \!H\rangle \in T\}$. Assume for reductio that $h \notin {\mathsf {H}}(T)$. Then there must be some transition $\langle m'\!\rightarrowtail \!H'\rangle \in T$ such that $h \notin H'$. Since by assumption *T* does not contain a maximal element, there is some transition $\langle m''\!\rightarrowtail \!H''\rangle \in T$ such that $\langle m'\!\rightarrowtail \!H'\rangle \prec \langle m''\!\rightarrowtail \!H''\rangle$. This implies that $H'' \subsetneq H'$ and hence $m'<m''$, from which it follows that ${\mathsf {H}}_{m''} \subseteq H' = [h']_{m'}$ for some $h' \in H'' \subsetneq {\mathsf {H}}_{m''}$. Since $m'' \in \{m\in M\mid \langle m\!\rightarrowtail \!H\rangle \in T\} \subseteq h$, we have that $h \in {\mathsf {H}}_{m''} \subseteq H'$, which contradicts our assumption that $h \notin H'$. $\square$

With the ordering relation $\prec$ on $\mathsf {trans}(\mathcal {M})$ at hand, we can close a set of transitions $T \subseteq \mathsf {trans}(\mathcal {M})$ downwards. A set of transitions *T* is said to be *downward closed* if it contains all transitions preceding any transition occurring in it as well. We call the set that results from closing a given transition set *T* downwards the *downward completion ofT*.

#### **Definition 9**

(*Downward closed*) For $\mathcal {M}=\langle M,<\rangle$ a BT structure, a transition set $T \subseteq \mathsf {trans}(\mathcal {M})$ is *downward closed* iff for all $t, t' \in \mathsf {trans}(\mathcal {M})$, if $t \in T$ and $t' \prec t$, then $t' \in T$.

#### **Definition 10**

(*Downward completion*) For $\mathcal {M}=\langle M,<\rangle$ a BT structure and $T \subseteq \mathsf {trans}(\mathcal {M})$ a set of transitions, the *downward completion ofT*, in symbols: ${{\mathsf {d}}}{{\mathsf {c}}}(T)$, is defined as follows:

$$\begin{aligned} {{\mathsf {d}}}{{\mathsf {c}}}(T)= \{t \in \mathsf {trans}(\mathcal {M})\mid \text {there is some }t' \in T\text { s.t. } t = t'\text { or }t \prec t'\}. \end{aligned}$$

Due to the absence of backward branching, the downward completion ${{\mathsf {d}}}{{\mathsf {c}}}(T)$ of a transition set *T* is uniquely determined. Moreover, the set of histories allowed by the downward completion ${{\mathsf {d}}}{{\mathsf {c}}}(T)$ of a transition set *T* is identical to the set of histories allowed by the set *T* itself, i.e., ${\mathsf {H}}(T)= {\mathsf {H}}({{\mathsf {d}}}{{\mathsf {c}}}(T))$. Closing a transition set downward does not exclude any histories. From this it follows immediately that the downward completion ${{\mathsf {d}}}{{\mathsf {c}}}(T)$ of a transition set *T* is consistent if and only if *T* is consistent.^7^

By Proposition 1, a consistent, downward closed transition set *T* is a $\prec$-chain of transitions that is closed toward the past. Unlike a history, which is a maximal $<$-chain in *M* and as such represents a complete possible course of events, a consistent, downward closed transition set is a possibly non-maximal $\prec$-chain in $\mathsf {trans}(\mathcal {M})$. As a possibly non-maximal $\prec$-linear set of transitions that is complete with respect to the past, a consistent, downward closed transition set uniquely captures a complete or incomplete possible course of events that stretches linearly from the past toward a possibly open future. Different consistent, downward closed sets of transitions correspond to different courses of events. The downward completion reflects the idea that the past is fixed and ensures that every possible consistent extension of a given transition set is a future extension. In case a consistent, downward closed set of transitions is a non-maximal $\prec$-chain in $\mathsf {trans}(\mathcal {M})$, it allows more than one history, and the possible course of events it represents is an incomplete one, viz. one that allows for alternative possible future continuations. Formally, every consistent, downward closed set of transitions *T* in a BT structure $\mathcal {M}=\langle M,<\rangle$ corresponds one-to-one to a subtree of $\mathcal {M}$ with domain $\bigcup {\mathsf {H}}(T)$, which comprises at least one history from $\mathsf {hist}(\mathcal {M})$ and is such that if it contains a branching point, it contains all moments $m \in M$ above that branching point as well. It should also be noted that a consistent, downward closed set of transitions does not have to contain a greatest element, i.e., not every consistent, downward closed set of transitions is identical to the downward completion of the singleton of a particular transition.

Given a BT structure $\mathcal {M}=\langle M,<\rangle$, we can define for any moment $m \in M$ some downward closed transition set that contains all transitions in the past of *m*. For $m \in M$, the *set of transitions precedingm*, ${{\mathsf {T}}}{{\mathsf {r}}}(m)$, is the set of all transitions whose outcome includes the set ${\mathsf {H}}_m$ of histories containing *m*. Obviously, ${{\mathsf {T}}}{{\mathsf {r}}}(m)$ is consistent, according to Definition 7. The set ${{\mathsf {T}}}{{\mathsf {r}}}(m)$ captures the past course of events up to the moment *m*, and we have that ${\mathsf {H}}({{\mathsf {T}}}{{\mathsf {r}}}(m)) = {\mathsf {H}}_m$. Note also that for $\langle m'\!\rightarrowtail \!H'\rangle \in \mathsf {trans}(\mathcal {M})$, ${\mathsf {H}}_m \subseteq H'$ implies that $m'<m$.

#### **Definition 11**

(*The set of transitions precedingm*) Given a BT structure $\mathcal {M}=\langle M,<\rangle$ and a moment $m \in M$, the *set of transitions precedingm*, in symbols: ${{\mathsf {T}}}{{\mathsf {r}}}(m)$, is defined as follows:

$$\begin{aligned} {{\mathsf {T}}}{{\mathsf {r}}}(m)= \{\langle m'\!\rightarrowtail \!H'\rangle \in \mathsf {trans}(\mathcal {M})\mid {\mathsf {H}}_m \subseteq H'\}. \end{aligned}$$

A set of transitions $T \subseteq \mathsf {trans}(\mathcal {M})$ is *maximal consistent* if it is consistent, but none of its proper supersets $T' \supsetneq T$ in $\mathsf {trans}(\mathcal {M})$ is. By Proposition 1, a maximal consistent transition set is a maximal $\prec$-chain in $\mathsf {trans}(\mathcal {M})$. The notion of a maximal consistent set of transitions provides the analogue to the notion of a history. While consistent sets of transitions can in general also capture incomplete courses of events, which correspond to non-singleton sets of histories, a maximal consistent set of transitions specifies exactly one history and thus describes a unique complete possible course of events. For any history $h \in \mathsf {hist}(\mathcal {M})$, we can define the *set of transitions characterizing the historyh*, ${{\mathsf {T}}}{{\mathsf {r}}}(h)$, as the subset of $\mathsf {trans}(\mathcal {M})$ that contains all and only those transitions that allow *h* to occur.

#### **Definition 12**

(*The set of transitions characterizingh*) Given a BT structure $\mathcal {M}=\langle M,<\rangle$ and a history $h \in \mathsf {hist}(\mathcal {M})$, the *set of transitions characterizingh*, in symbols: ${{\mathsf {T}}}{{\mathsf {r}}}(h)$, is defined as follows:

$$\begin{aligned} {{\mathsf {T}}}{{\mathsf {r}}}(h)= \{\langle m\!\rightarrowtail \!H\rangle \in \mathsf {trans}(\mathcal {M})\mid h \in H\}. \end{aligned}$$

By Definition 7 it immediately follows that ${{\mathsf {T}}}{{\mathsf {r}}}(h)$ is consistent since $h \in {\mathsf {H}}({{\mathsf {T}}}{{\mathsf {r}}}(h))$. The set ${{\mathsf {T}}}{{\mathsf {r}}}(h)$ is even maximal consistent and thus, in particular, downward closed.

#### **Proposition 2**

Let $\mathcal {M}=\langle M,<\rangle$ be a BT structure and $h \in \mathsf {hist}(\mathcal {M})$ a history. The set ${{\mathsf {T}}}{{\mathsf {r}}}(h)$ of transitions characterizing the history *h* is maximal consistent.

#### *Proof*

Obviously, ${{\mathsf {T}}}{{\mathsf {r}}}(h)$ is consistent, since $h \in {\mathsf {H}}({{\mathsf {T}}}{{\mathsf {r}}}(h))$. We show that ${{\mathsf {T}}}{{\mathsf {r}}}(h)$ is maximal consistent. Let $\langle m\!\rightarrowtail \!H\rangle \in \mathsf {trans}(\mathcal {M})$ be a transition s.t. $\langle m\!\rightarrowtail \!H\rangle \not \in {{\mathsf {T}}}{{\mathsf {r}}}(h)$, i.e., $h \notin H$. Then there is some history $h' \in H$ s.t. $h \not \equiv _m h'$. By Definitions 1 and 4 it follows that there is some moment $m' \in M$ s.t. $m' \le m$ and $h \perp _{m'} h'$. Since $H = [h']_m \!\subseteq \! [h']_{m'}$ and $\langle m'\!\rightarrowtail \![h]_{m'}\rangle \!\in \!{{\mathsf {T}}}{{\mathsf {r}}}(h)$, we have that ${\mathsf {H}}({{\mathsf {T}}}{{\mathsf {r}}}(h)\cup \{\langle m\!\rightarrowtail \!H\rangle \}) \!=\! \emptyset$.

$$\square $$

In fact, every maximal consistent transition set *T* allows exactly one history $h \in \mathsf {hist}(\mathcal {M})$, i.e., ${\mathsf {H}}(T)= \{h\}$, and is identical to a set of transitions ${{\mathsf {T}}}{{\mathsf {r}}}(h)$.

#### **Proposition 3**

Let $\mathcal {M}=\langle M,<\rangle$ be a BT structure and $T \subseteq \mathsf {trans}(\mathcal {M})$ a maximal consistent set of transitions. Then there is some history $h \in \mathsf {hist}(\mathcal {M})$ s.t. (i) ${\mathsf {H}}(T)= \{h\}$ and (ii) $T = {{\mathsf {T}}}{{\mathsf {r}}}(h)$.

#### *Proof*

(i) Since by assumption ${\mathsf {H}}(T)\ne \emptyset$, there is at least one history $h \in {\mathsf {H}}(T)$. Assume for reductio that there is another history $h' \ne h$ s.t. ${\mathsf {H}}(T)\supseteq \{h,h'\}$. By Definitions 1 and 4 it follows that there is some moment $m \in M$ s.t. $h \perp _m h'$ and thus ${\varPi }_m \supseteq \{[h]_m,[h']_m\}$. We show that $\langle m\!\rightarrowtail \![h]_m\rangle \not \in T$, but ${\mathsf {H}}(T \cup \{\langle m\!\rightarrowtail \![h]_m\rangle \}) \ne \emptyset$. Assume that $\langle m\!\rightarrowtail \![h]_m\rangle \in T$. Then ${\mathsf {H}}(T)\subseteq [h]_m$. Since $h' \notin [h]_m$, it follows that $h' \not \in {\mathsf {H}}(T)$, which contradicts our assumption that ${\mathsf {H}}(T)\supseteq \{h,h'\}$. Therefore, $\langle m\!\rightarrowtail \![h]_m\rangle \notin T$. Since $h \in {\mathsf {H}}(T)\cap [h]_m$, it nevertheless holds that ${\mathsf {H}}(T \cup \{\langle m\!\rightarrowtail \![h]_m\rangle \}) \ne \emptyset$. This contradicts the maximal consistency of *T*.
(ii) By (i), ${\mathsf {H}}(T)= \{h\}$. Thus, $T \subseteq {{\mathsf {T}}}{{\mathsf {r}}}(h)$. We show that ${{\mathsf {T}}}{{\mathsf {r}}}(h)\subseteq T$. Assume for reductio that there is some transition $\langle m\!\rightarrowtail \!H\rangle \in {{\mathsf {T}}}{{\mathsf {r}}}(h)$ but $\langle m\!\rightarrowtail \!H\rangle \notin T$. It follows that $h \in {\mathsf {H}}(T)\cap H$ and thus ${\mathsf {H}}(T \cup \{\langle m\!\rightarrowtail \!H\rangle \}) \ne \emptyset$. This contradicts the maximal consistency of *T*.$\square$

There is thus a natural one-to-one correspondence between maximal consistent sets of transitions and histories: every maximal consistent set of transitions $T \subseteq \mathsf {trans}(\mathcal {M})$ allows exactly one history $h \in \mathsf {hist}(\mathcal {M})$ and is of the form ${{\mathsf {T}}}{{\mathsf {r}}}(h)$; and for every history $h \in \mathsf {hist}(\mathcal {M})$, there is some maximal consistent set of transitions ${{\mathsf {T}}}{{\mathsf {r}}}(h)$, which characterizes that history.

### BT Semantics with Sets of Transitions

The transition language ${\mathscr {L}}_{{\mathsf {t}}}$ extends the propositional language ${\mathscr {L}}$ by the following operators: a future operator ${\mathsf {F}}$ and a past operator ${\mathsf {P}}$, an operator for inevitability $\Box$ and a stability operator ${\mathsf {S}}$. As mentioned, the basic idea is to replace the moment-history pairs employed in the Ockhamist semantics by pairs consisting of a moment and a consistent, downward closed set of transitions. For $\mathcal {M}=\langle M,<\rangle$ a BT structure, let $\mathsf {dcts}(\mathcal {M})$ be the *set of all consistent, downward closed sets of transitions* in $\mathcal {M}$, which includes at least the empty transition set that is denoted by $\mathsf {\emptyset _{Tr}}$. That is, the set $\mathsf {dcts}(\mathcal {M})$ contains for every transition set $T \subseteq \mathsf {trans}(\mathcal {M})$ that is consistent, its downward completion ${{\mathsf {d}}}{{\mathsf {c}}}(T)$; so $\mathsf {dcts}(\mathcal {M})= \{{{\mathsf {d}}}{{\mathsf {c}}}(T)\mid T \subseteq \mathsf {trans}(\mathcal {M})\text { and } {\mathsf {H}}(T)\ne \emptyset \}$. The set $\mathsf {dcts}(\mathcal {M})$ provides the full range of transition sets that will be taken into account in the semantic evaluation on a BT structure $\mathcal {M}=\langle M,<\rangle$. Each consistent, downward closed set of transitions corresponds one-to-one to a complete or incomplete possible course of events that stretches linearly from the past toward a possibly open future. The restriction of the semantic evaluation to downward closed transition sets reflects the idea that the past is fixed and ensures that every possible consistent extension of a given transition set affects the future and excludes at least one history.^8^

In order for a pair consisting of a moment $m \in M$ and a consistent, downward closed set of transitions $T \in \mathsf {dcts}(\mathcal {M})$ to constitute a suitable index of evaluation, the transition set *T* must allow at least one history that contains the moment of evaluation $m \in M$, i.e., ${\mathsf {H}}(T)\cap {\mathsf {H}}_m \ne \emptyset$. In other words, the moment of evaluation $m \in M$ must be *compatible* with the transition set $T \in \mathsf {dcts}(\mathcal {M})$. In analogy with the Ockhamist case, we employ the notation “*m* / *T*” in order to indicate that the condition is met. We now provide the semantic clauses for the temporal operators ${\mathsf {F}}$ and ${\mathsf {P}}$, the inevitability operator $\Box$ and the stability operator ${\mathsf {S}}$. We use ${\mathfrak {M}},m/T \vDash _{{\mathsf {t}}}\phi$ in order to indicate that a sentence $\phi \in {\mathscr {L}}_{{\mathsf {t}}}$ is true at a pair *m* / *T* in a transition model ${\mathfrak {M}}= \langle \mathcal {M},v_{\mathsf {t}}\rangle$ on a BT structure $\mathcal {M}=\langle M,<\rangle$ with $v_{\mathsf {t}}: {{\mathsf {A}}}{{\mathsf {t}}}{\times }\{m/T\mid m \in M\text {, } T \in \mathsf {dcts}(\mathcal {M})\text { and } {\mathsf {H}}(T)\cap {\mathsf {H}}_m \ne \emptyset \} \rightarrow \{0,1\}$ according to the transition semantics.^9^

#### Temporal Operators

In the case of the temporal operators ${\mathsf {F}}$ and ${\mathsf {P}}$, the transition set $T \in \mathsf {dcts}(\mathcal {M})$ is kept fixed, and the moment of evaluation $m \in M$ is shifted in a way compatible with that transition set. As said, the crucial point in developing a propositional semantics on a BT structure consists in spelling out appropriate truth conditions for the future operator. The future operator ${\mathsf {F}}$ of the transition semantics has both Peircean and Ockhamist traits: it combines the idea of universally quantifying over future possibilities with the Ockhamist idea of relativizing truth to a possible course of events. Unlike in Ockhamism, the possible course of events needs not to be a complete one, however; and whereas the Peircean future operator requires a witness in every possible future continuation of the moment of evaluation, our future operator demands a witness in only those future continuations of the moment of evaluation that are possible extensions of the given transition set. Along those lines, we can say that a sentence of the form ${\mathsf {F}}\phi$ is true at an index *m* / *T* iff for every extension $T' \supseteq T$ that is compatible with the moment of evaluation *m*, there is a compatible future moment $m'>m$ at which $\phi$ is true with respect to the original transition set.

($${\mathsf {F}}^\sharp $$)
$${\mathfrak {M}},m/T \vDash _{{\mathsf {t}}}{\mathsf {F}}\phi \text { iff for all } T' \supseteq T \text { s.t. } {\mathsf {H}}(T') \cap {\mathsf {H}}_m \ne \emptyset \text {, there is some } m'>m \text { s.t.}$$$${\mathsf {H}}(T')\cap {\mathsf {H}}_{m'} \ne \emptyset \text { and } {\mathfrak {M}},m'/T \vDash _{{\mathsf {t}}}\phi $$.

Note that the sole function of the universal quantification over the possible future extensions $T'$ of the given transition set *T* is to specify the range of possible future continuations of *m* that are required to contain a witness for the future claim. The semantic clause (${\mathsf {F}}^\sharp$) is equivalent to the following condition, in which the universal quantification over future possibilities is spelled out by reference to the set of histories allowed by the given transition set rather than in terms of its possible future extensions:

($${\mathsf {F}}$$)
$${\mathfrak {M}},m/T \vDash _{{\mathsf {t}}}{\mathsf {F}}\phi \text { iff for all } h \in {\mathsf {H}}(T)\cap {\mathsf {H}}_m \text {, there is some } m' \in h \text { s.t. } m'>m \text { and}$$$${\mathfrak {M}},m'/T \vDash _{{\mathsf {t}}}\phi $$.^10^

Obviously, (${\mathsf {F}}^\sharp$) implies (${\mathsf {F}})$: if for every $T' \supseteq T$ s.t. ${\mathsf {H}}(T')\cap {\mathsf {H}}_m \ne \emptyset$, there is a future witness $m'>m$ s.t. ${\mathsf {H}}(T')\cap {\mathsf {H}}_{m'} \ne \emptyset$, then this holds, in particular, for every maximal consistent extension $T'$ of *T*. Since by Proposition 2 for every $h \in {\mathsf {H}}(T)\cap {\mathsf {H}}_m$, there is a maximal consistent extension ${{\mathsf {T}}}{{\mathsf {r}}}(h)\supsetneq T$ with ${\mathsf {H}}({{\mathsf {T}}}{{\mathsf {r}}}(h)) = \{h\}$, condition (${\mathsf {F}}^\sharp$) implies the existence of a future witness $m'>m$ in every history $h \in {\mathsf {H}}(T)\cap {\mathsf {H}}_m$. On the other hand, let $T' \supseteq T$ be an extension of *T* s.t. ${\mathsf {H}}(T')\cap {\mathsf {H}}_m \ne \emptyset$. Given that (${\mathsf {F}}$) holds, for every $h \in {\mathsf {H}}(T')\cap {\mathsf {H}}_m \subseteq {\mathsf {H}}(T)\cap {\mathsf {H}}_m$, there is some future witness $m'>m$ in *h* so that ${\mathsf {H}}(T')\cap {\mathsf {H}}_{m'} \ne \emptyset$. Since (${\mathsf {F}}$) is equivalent with (${\mathsf {F}}^\sharp$) and easier to grasp, we will make use of (${\mathsf {F}}$) in what follows. We will thus say that a sentence of the form ${\mathsf {F}}\phi$ is true at an index of evaluation *m* / *T* if and only if every history that passes through the moment *m* and is admitted by *T* contains some later moment $m'$ at which $\phi$ is true with respect to *T*.^11^

The semantic clause for the past operator ${\mathsf {P}}$ is straightforward: a sentence of the form ${\mathsf {P}}\phi$ is true at an index of evaluation *m* / *T* if and only if there is some earlier moment $m'<m$ such that $\phi$ is true at $m'$ with respect to *T*. Due to the absence of backward branching, every moment has a unique past, and given that the moment of evaluation is compatible with the given transition set, all moments in its past are so as well, so that no further specification is needed in that case.

($${\mathsf {P}}$$)
$${\mathfrak {M}},m/T \vDash _{{\mathsf {t}}}{\mathsf {P}}\phi \text { iff there is some } m'<m \text { s.t. } {\mathfrak {M}},m'/T \vDash _{{\mathsf {t}}}\phi $$.

#### Modal Operators

Since—like in Ockhamism but unlike in Peirceanism—the semantic evaluation is relativized to a second parameter of truth, which specifies some possible course of events, modal operators are interpretable, and truth and inevitability come apart. In the case of the modal operators, the moment of evaluation $m \in M$ is kept constant, and the modalities are interpreted as quantifiers over all transition sets $T \in \mathsf {dcts}(\mathcal {M})$ that are compatible with that moment *m*. The inevitability operator $\Box$ amounts to universal quantification. A modal operator for possibility, $\Diamond$, can be defined as its dual, i.e., $\Diamond := \lnot \Box \lnot$. As in standard Ockhamism, the modality involved is ${{\mathsf {S}}}{{\mathsf {5}}}$. The truth of a sentence of the form $\Box \phi$ with respect to some index of evaluation *m* / *T* can be understood along the following lines: relative to any—complete or incomplete—possible course of events compatible with *m*, $\phi$ is true at *m*. Note that, unlike in Ockhamism, an incomplete possible course of events suffices as a witness for a possibility claim.

($$\Box $$)
$${\mathfrak {M}},m/T \vDash _{{\mathsf {t}}}\Box \phi \text { iff for all } T' \in \mathsf {dcts}(\mathcal {M})\text { s.t. } {\mathsf {H}}(T')\cap {\mathsf {H}}_m \ne \emptyset \text {, } {\mathfrak {M}},m/T' \vDash _t \phi $$.

#### Stability Operators

Evaluating sentences with respect to consistent, downward closed sets of transitions rather than with respect to entire histories brings in a new phenomenon that is specific to the transition approach. The truth value of a sentence at a moment can change if the transition set is extended so that it stretches further into the future. Next to temporal and modal operators, the transition semantics allows for a stability operator, ${\mathsf {S}}$, which is interpreted as a universal quantifier over the possible extensions of a given transition set that are compatible with the moment of evaluation. The stability operator ${\mathsf {S}}$ enables us to specify how and how far time has to unfold for the truth value of a sentence at a moment to become settled, or stable, as we will say. The dual  of the stability operator, an existential quantifier over the possible extensions of a transition set, can be defined as follows: . The modality involved is ${{\mathsf {S}}}{{\mathsf {4}}}$. A sentence of the form ${\mathsf {S}}\phi$ is true at an index of evaluation *m* / *T* iff $\phi$ is true at *m* with respect to any possible extension $T'$ of *T* that is compatible with *m*. Given that ${\mathsf {S}}\phi$ is true with respect to *m* / *T*, whatever else will happen, i.e. no matter how we extend the transition set *T*, $\phi$ remains true at *m*.

($${\mathsf {S}}$$)
$${\mathfrak {M}},m/T \vDash _{{\mathsf {t}}}{\mathsf {S}}\phi \text { iff for all } T' \supseteq T \text { s.t. } {\mathsf {H}}(T')\cap {\mathsf {H}}_m \ne \emptyset , {\mathfrak {M}},m/T' \vDash _{{\mathsf {t}}}\phi $$.

If in a transition model ${\mathfrak {M}}$, the sentence ${\mathsf {S}}\phi$ is true at an index of evaluation *m* / *T*, i.e., ${\mathfrak {M}},m/T \vDash _{{\mathsf {t}}}{\mathsf {S}}\phi$, we say that $\phi$ is *stably-true* relative to that index. Accordingly, we say that $\phi$ is *stably-false* relative to an index of evaluation *m* / *T* if its negation $\lnot \phi$ is stably-true at that index, i.e., ${\mathfrak {M}},m/T \vDash _{{\mathsf {t}}}{\mathsf {S}}\lnot \phi$.

There are sentences that classify as neither stably-true nor stably-false with respect to a given index of evaluation *m* / *T*: those sentences are said to be *contingent* relative to *m* / *T*. They are true at the moment *m* with respect to one extension of *T* that is compatible with *m* but false with respect to another. In other words, a sentence $\phi$ is contingent relative to *m* / *T* if and only if  is true at *m* / *T*, i.e.,  or, equivalently, ${\mathfrak {M}},m/T \vDash _{{\mathsf {t}}}\lnot {\mathsf {S}}\phi \wedge \lnot {\mathsf {S}}\lnot \phi$.

A sentence $\phi$ that is contingent relative to some index of evaluation *m* / *T* can become stably-true or stably-false with respect to some extension $T'$ of the transition set *T*. Once the truth value of the sentence $\phi$ has become stable with respect to some index, however, it remains stable under all extensions of the transition set in question. In particular, a sentence that is stably-true with respect to some index *m* / *T* remains stably-true at *m* relative to all extensions $T' \supseteq T$ compatible with *m*.

The domain of quantification of the stability operator ${\mathsf {S}}$ is only a subset of the domain of quantification of the inevitability operator $\Box$. Whereas the inevitability operator quantifies over all transition sets compatible with the moment of evaluation, the stability operator quantifies over only the possible future extensions of the given transition set. Inevitability implies stability, but not *vice versa*. That the truth of a sentence $\phi$ is inevitable at an index *m* / *T* means that the sentence $\phi$ is true at the moment *m* no matter what happens. That a sentence $\phi$ is stably-true relative to an index *m* / *T*, on the other hand, expresses that given the course of events specified by the transition set *T*, it is settled that $\phi$ is true at the moment *m*: with respect to that course of events, the sentence $\phi$ is true at the moment *m* no matter what will happen later on. As a universal quantifier over the possible future extensions of a given course of events, the stability operator allows for a perspicuous treatment of sentences about the future whose truth value at a moment only stabilizes as the future unfolds. It enables us to capture the behavior of the truth value of a sentence about the future at a moment in the course of time, viz. its changing from contingent to stably-true or stably-false. We will come back to the role of the stability operator in the context of future contingents in Sect. 4.4 below.

### Sentences About the Future in the Transition Semantics

**Fig. 1:**
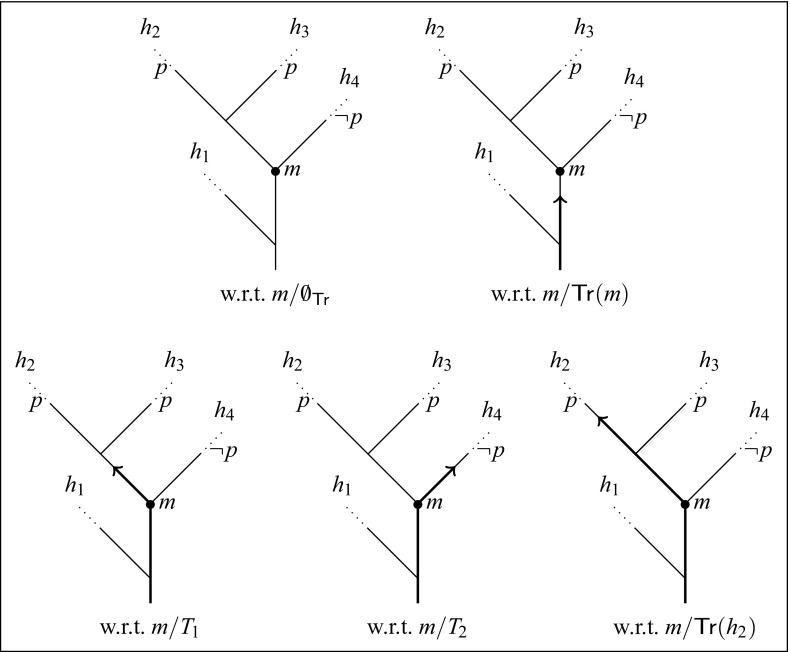
Evaluation w.r.t. different transition sets

In the transition semantics, sentences are evaluated at a moment with respect to a possible course of events compatible with that moment, just as in Ockhamism. The crucial difference between the transition semantics and Ockhamism consists in the fact that whereas the histories employed in the Ockhamist semantics represent complete possible courses of events, the transition semantics allows for the relativization to incomplete possible courses of events as well. In order to illustrate what that difference amounts to, let us have a look at how sentences about the future are treated in the transition semantics. For that purpose, consider a model ${\mathfrak {M}}= \langle \mathcal {M},v_{\mathsf {t}}\rangle$ on a BT structure $\mathcal {M}=\langle M,<\rangle$ that contains a branching point *m* such that there is one possible future continuation of *m* in which *p* always is the case and another in which *p* never is the case, where the truth value of *p* is only moment-dependent. That is, in the model in Fig. 1, we assume that for all *T* s.t. ${\mathsf {H}}(T)\cap {\mathsf {H}}_m \ne \emptyset$, $v_{\mathsf {t}}(p,m'/T) = 1$ if $m'>m$ and $m' \in h_2 \cup h_3$, and $v_{\mathsf {t}}(p,m'/T) = 0$ if $m'>m$ and $m' \in h_4$. We will now investigate the behavior of the truth value of the sentence ${\mathsf {F}}p$ and its contrary prediction ${\mathsf {F}}\lnot p$ at the moment *m* with respect to different transition sets, as indicated in Fig. 1.

Let us start with the empty transition set, $\mathsf {\emptyset _{Tr}}$, which excludes none of the histories passing through the moment *m*, since ${\mathsf {H}}(\mathsf {\emptyset _{Tr}}) = \mathsf {hist}(\mathcal {M})$. If we evaluate the sentences ${\mathsf {F}}p$ and ${\mathsf {F}}\lnot p$ at the index $m/\mathsf {\emptyset _{Tr}}$, both sentences turn out false, i.e., ${\mathfrak {M}},m/\mathsf {\emptyset _{Tr}}\not \vDash _{{\mathsf {t}}}{\mathsf {F}}p$ and ${\mathfrak {M}},m/\mathsf {\emptyset _{Tr}}\not \vDash _{{\mathsf {t}}}{\mathsf {F}}\lnot p$. The sentence ${\mathsf {F}}p$ is false at $m/\mathsf {\emptyset _{Tr}}$ since the empty transition set $\mathsf {\emptyset _{Tr}}$ admits the history $h_4 \in {\mathsf {H}}_m$, in which *p* is false at any moment later than *m*; and ${\mathsf {F}}\lnot p$ is equally false at $m/\mathsf {\emptyset _{Tr}}$ since the empty transition set $\mathsf {\emptyset _{Tr}}$ also allows the history $h_2 \in {\mathsf {H}}_m$, in which *p* holds at any moment later than *m*. The same is true if we evaluate the sentences at the moment *m* with respect to the set of transitions preceding *m*, ${{\mathsf {T}}}{{\mathsf {r}}}(m)$, as ${\mathsf {H}}(\mathsf {\emptyset _{Tr}}) \cap {\mathsf {H}}_m = {\mathsf {H}}({{\mathsf {T}}}{{\mathsf {r}}}(m)) \cap {\mathsf {H}}_m$ although $h_1 \in {\mathsf {H}}(\mathsf {\emptyset _{Tr}})\setminus {\mathsf {H}}({{\mathsf {T}}}{{\mathsf {r}}}(m))$. Again, both the sentence ${\mathsf {F}}p$ and its contrary prediction ${\mathsf {F}}\lnot p$ turn out false at the index $m/{{\mathsf {T}}}{{\mathsf {r}}}(m)$, i.e., ${\mathfrak {M}},m/{{\mathsf {T}}}{{\mathsf {r}}}(m)\not \vDash _{{\mathsf {t}}}{\mathsf {F}}p$ and ${\mathfrak {M}},m/{{\mathsf {T}}}{{\mathsf {r}}}(m)\not \vDash _{{\mathsf {t}}}{\mathsf {F}}\lnot p$. With both the sentences ${\mathsf {F}}p$ and ${\mathsf {F}}\lnot p$ being false at $m/\emptyset$ and $m/{{\mathsf {T}}}{{\mathsf {r}}}(m)$, respectively, their disjunction ${\mathsf {F}}p \vee {\mathsf {F}}\lnot p$ is likewise false at those indices, i.e., ${\mathfrak {M}},m/\mathsf {\emptyset _{Tr}}\not \vDash _{{\mathsf {t}}}{\mathsf {F}}p \vee {\mathsf {F}}\lnot p$ and ${\mathfrak {M}},m/{{\mathsf {T}}}{{\mathsf {r}}}(m)\not \vDash _{{\mathsf {t}}}{\mathsf {F}}p \vee {\mathsf {F}}\lnot p$. Yet, the disjunction is false at those indices only contingently, as we shall see.

With respect to the transition set $T_1$, which specifies the immediate possible future continuation of *m* in which *p* always is the case, the sentence ${\mathsf {F}}p$ is true at the moment *m*, while its contrary prediction is false at that index, i.e., ${\mathfrak {M}},m/T_1 \vDash _{{\mathsf {t}}}{\mathsf {F}}p$ and ${\mathfrak {M}},m/T_1 \not \vDash _{{\mathsf {t}}}{\mathsf {F}}\lnot p$. The transition set $T_1$ excludes any history in ${\mathsf {H}}_m$ that lacks a witness for the future claim ${\mathsf {F}}p$. With respect to the transition set $T_2$, on the other hand, which captures the immediate possible future continuation of *m* in which *p* never is the case, ${\mathsf {F}}\lnot p$ is true at *m*, while ${\mathsf {F}}p$ is false at that index, i.e., ${\mathfrak {M}},m/T_2 \vDash _{{\mathsf {t}}}{\mathsf {F}}\lnot p$ and ${\mathfrak {M}},m/T_2 \not \vDash _{{\mathsf {t}}}{\mathsf {F}}p$. Since there is a transition set compatible with *m* with respect to which ${\mathsf {F}}p$ is true and ${\mathsf {F}}\lnot p$ is false at *m* and another with respect to which ${\mathsf {F}}\lnot p$ is true and ${\mathsf {F}}p$ is false at *m*, each of the possibility claims $\Diamond {\mathsf {F}}p$, $\Diamond \lnot {\mathsf {F}}\lnot p$, $\Diamond {\mathsf {F}}\lnot p$ and $\Diamond \lnot {\mathsf {F}}p$ is true at the moment *m* with respect to any transition set compatible with that moment. In particular, we have that at the index $m/T_1$ both ${\mathsf {F}}p$ and $\Diamond \lnot {\mathsf {F}}p$ are true, i.e., ${\mathfrak {M}},m/T_1 \vDash _{{\mathsf {t}}}{\mathsf {F}}p \wedge \Diamond \lnot {\mathsf {F}}p$, which shows that future truth and inevitability come apart, as they should. Note that ${\mathsf {F}}p$ is obviously also true at *m* with respect to the transition set ${{\mathsf {T}}}{{\mathsf {r}}}(h_2)$, which is maximal consistent and characterizes the history $h_2$. Since we have ${\mathfrak {M}},m/{{\mathsf {T}}}{{\mathsf {r}}}(h_2) \vDash _{{\mathsf {t}}}{\mathsf {F}}p$, the transition set ${{\mathsf {T}}}{{\mathsf {r}}}(h_2)$ constitutes a witness for the possibility claim $\Diamond {\mathsf {F}}p$ at the moment *m*, just as the transition set $T_1$ does. Yet in contrast to ${{\mathsf {T}}}{{\mathsf {r}}}(h_2)$, the transition set $T_1$ provides a *local* witness for that possibility: the possibility ${\mathsf {F}}p$ arises at the branching point *m*, even though the relevant element of the partition contains more than one history.

Since both $T_1$ and $T_2$ are extensions of the transition set ${{\mathsf {T}}}{{\mathsf {r}}}(m)$, with ${\mathsf {F}}p$ being true at $m/T_1$ and false at $m/T_2$, we have that  is true at *m* with respect to the set ${{\mathsf {T}}}{{\mathsf {r}}}(m)$ of transitions preceding that moment, i.e.,  or, equivalently, ${\mathfrak {M}},m/{{\mathsf {T}}}{{\mathsf {r}}}(m)\vDash _{{\mathsf {t}}}\lnot {\mathsf {S}}{\mathsf {F}}p \wedge \lnot {\mathsf {S}}\lnot {\mathsf {F}}p$. The sentence ${\mathsf {F}}p$ is contingent with respect to $m/{{\mathsf {T}}}{{\mathsf {r}}}(m)$: it is neither stably-true nor stably-false relative to that index. With respect to the transition set $T_1$, and likewise $T_2$, however, the sentence ${\mathsf {F}}p$ is not contingent anymore at the moment *m*. Its truth value at the moment *m* stabilizes with respect to any possible extension of ${{\mathsf {T}}}{{\mathsf {r}}}(m)$: the sentence ${\mathsf {F}}p$ is stably-true with respect to $m/T_1$ and stably-false with respect to $m/T_2$, i.e., ${\mathfrak {M}},m/T_1 \vDash _{{\mathsf {t}}}{\mathsf {S}}{\mathsf {F}}p$ and ${\mathfrak {M}},m/T_2 \vDash _{{\mathsf {t}}}{\mathsf {S}}\lnot {\mathsf {F}}p$. The sentence ${\mathsf {F}}p$ is true at the index $m/T_1$ and remains true at *m* with respect to every proper extension of $T_1$, such as ${{\mathsf {T}}}{{\mathsf {r}}}(h_2)$. Being stably-true at *m* with respect to $T_1$, ${\mathsf {F}}p$ is also stably-true at *m* with respect to ${{\mathsf {T}}}{{\mathsf {r}}}(h_2) \supsetneq T_1$, i.e., ${\mathfrak {M}},m/{{\mathsf {T}}}{{\mathsf {r}}}(h_2) \vDash _{{\mathsf {t}}}{\mathsf {S}}{\mathsf {F}}p$. Neither at $m/T_1$ nor at $m/{{\mathsf {T}}}{{\mathsf {r}}}(h_2)$ is it inevitable, however, that ${\mathsf {F}}p$. The sentence $\Box {\mathsf {F}}p$ is false at both those indices, i.e., ${\mathfrak {M}},m/T_1 \not \vDash _{{\mathsf {t}}}\Box {\mathsf {F}}p$ and ${\mathfrak {M}},m/{{\mathsf {T}}}{{\mathsf {r}}}(h_2) \not \vDash _{{\mathsf {t}}}\Box {\mathsf {F}}p$, since ${\mathfrak {M}},m/T_2 \not \vDash _{{\mathsf {t}}}{\mathsf {F}}p$. A sentence can be stably-true relative to an index of evaluation without its truth at that index being inevitable.

We said that the sentence ${\mathsf {F}}p$ is contingent at the moment *m* with respect to the set ${{\mathsf {T}}}{{\mathsf {r}}}(m)$ of transitions preceding that moment. Accordingly, it is also contingent at *m* with respect to any proper subset of ${{\mathsf {T}}}{{\mathsf {r}}}(m)$, such as the empty transition set $\mathsf {\emptyset _{Tr}}$. Its truth value at the moment *m* depends on how the future unfolds and stabilizes only as time progresses. The same is true for ${\mathsf {F}}\lnot p$ and hence for the disjunction ${\mathsf {F}}p \vee {\mathsf {F}}\lnot p$. At the moment *m*, we have , since ${\mathfrak {M}},m/T_1 \vDash _{{\mathsf {t}}}{\mathsf {F}}p \vee {\mathsf {F}}\lnot p$ and ${\mathfrak {M}},m/T_2 \vDash _{{\mathsf {t}}}{\mathsf {F}}p \vee {\mathsf {F}}\lnot p$ but ${\mathfrak {M}},m/{{\mathsf {T}}}{{\mathsf {r}}}(m)\not \vDash _{{\mathsf {t}}}{\mathsf {F}}p \vee {\mathsf {F}}\lnot p$. That the disjunction is false at the moment *m* with respect to ${{\mathsf {T}}}{{\mathsf {r}}}(m)$ is a sign of contingency: the course of events up to *m* does not yet suffice to settle the matter. Only as the future unfolds, the truth value of either disjunct eventually stabilizes—in a maximal consistent extension, if not before—rendering the disjunction stably-true at the moment *m* as well. We have ${\mathfrak {M}},m/T_1 \vDash _{{\mathsf {t}}}{\mathsf {S}}({\mathsf {F}}p \vee {\mathsf {F}}\lnot p)$ and ${\mathfrak {M}},m/T_2 \vDash _{{\mathsf {t}}}{\mathsf {S}}({\mathsf {F}}p \vee {\mathsf {F}}\lnot p)$. The intuition that the disjunction ${\mathsf {F}}p \vee {\mathsf {F}}\lnot p$ has the force of a tautology, as expressed by Thomason (1970), is reflected in the transition semantics by the validity $\lnot {\mathsf {S}}\lnot ({\mathsf {F}}p \vee {\mathsf {F}}\lnot p)$ or, equivalently . The disjunction is never stably-false. As time progresses, sooner or later, things will become settled one way or the other.

## The Generality of the Transition Semantics

Every BT structure $\mathcal {M}=\langle M,<\rangle$ provides a set of moments *M*, a set of histories $\mathsf {hist}(\mathcal {M})$ and a set of transitions $\mathsf {trans}(\mathcal {M})$. Peirceanism, Ockhamism and the transition account differ with respect to which of those structural elements are employed as parameters of truth in the semantic evaluation. In this section we show that the transition semantics generalizes and properly extends both Peirceanism and Ockhamism. Being based on sets of transitions, the transition semantics exploits the full resources a BT structure has to offer. It thereby gains expressive means that are not available on either of those accounts, which are shown to make use of restricted means only. Zanardo (1998) likewise provides a framework that generalizes both Peirceanism and Ockhamism. We will discuss that framework in Sect. 4.4 below and illustrate to what extent the transition semantics with its stability operator exceeds also that account with respect to expressive strength.

In the transition semantics as outlined in Sect. 3.2, the parameters of truth employed in the semantic evaluation are provided by pairs consisting of a moment and a consistent, downward closed set of transitions. A sentence $\phi \in {\mathscr {L}}_{{\mathsf {t}}}$ is assigned a truth value in a model ${\mathfrak {M}}= \langle \mathcal {M},v_{\mathsf {t}}\rangle$ on a BT structure $\mathcal {M}=\langle M,<\rangle$ at a moment $m \in M$ with respect to a transition set $T \in \mathsf {dcts}(\mathcal {M})$ such that ${\mathsf {H}}(T)\cap {\mathsf {H}}_m \ne \emptyset$. A set of transitions $T \in \mathsf {dcts}(\mathcal {M})$ allows certain histories $h \in \mathsf {hist}(\mathcal {M})$ to occur and excludes others. There are two extreme cases. If $T \in \mathsf {dcts}(\mathcal {M})$ is the empty transition set, i.e., $T = \mathsf {\emptyset _{Tr}}$, it excludes no histories whatsoever. The set of histories allowed by the empty transition set $\mathsf {\emptyset _{Tr}}$ is the set of all histories in $\mathcal {M}$, i.e., ${\mathsf {H}}(\mathsf {\emptyset _{Tr}}) = \mathsf {hist}(\mathcal {M})$. If, on the other hand, $T \in \mathsf {dcts}(\mathcal {M})$ is a maximal consistent transition set, it excludes all but one history. By Proposition 3, every maximal consistent set of transitions is identical to a set ${{\mathsf {T}}}{{\mathsf {r}}}(h)$ that characterizes exactly one history $h \in \mathsf {hist}(\mathcal {M})$ and allows only that history to occur, i.e., ${\mathsf {H}}({{\mathsf {T}}}{{\mathsf {r}}}(h)) = \{h\}$. We show that if only the empty transition set $\mathsf {\emptyset _{Tr}}$ is taken into account in the semantic evaluation on a BT structure, we are back to Peirceanism, while a restriction to all maximal consistent transition sets yields Ockhamism.

### Transition Structures

In order to be able to capture the restriction of the semantic evaluation on a BT structure $\mathcal {M}=\langle M,<\rangle$ to subsets of $\mathsf {dcts}(\mathcal {M})$ in a uniform way, we generalize the notion of a transition model by introducing the notion of a *transition structure*. A transition structure $\mathcal {M}^{ ts } = \langle M,<, ts \rangle$ is a BT structure $\mathcal {M}=\langle M,<\rangle$ together with a non-empty set of transition sets $ts \subseteq \mathsf {dcts}(\mathcal {M})$ such that every moment $m \in M$ is compatible with at least one transition set $T \in ts$. The set $ts$ of transition sets is required to be such that it covers the entire set of moments, which figure as the fundamental elements of a BT structure.^12^ Note that the set $ts$ does not really ‘add’ anything to the BT structure: the BT structure itself already determines all possible sets of transitions in $\mathsf {dcts}(\mathcal {M})$. The set $ts$ merely indicates which of those transition sets are employed in the semantic evaluation. We define a transition model as a pair ${\mathfrak {M}}^{ ts } = \langle \mathcal {M}^{ ts },v_{\mathsf {t}}\rangle$, where $\mathcal {M}^{ ts } = \langle M,<, ts \rangle$ is a transition structure and $v_{\mathsf {t}}$ a valuation function that assigns truth values to the propositional variables of ${\mathscr {L}}_{{\mathsf {t}}}$ at a moment $m \in M$ relative to a transition set $T \in ts$ s.t. ${\mathsf {H}}(T)\cap {\mathsf {H}}_m \ne \emptyset$.

#### **Definition 13**

(*Transition structure*) A *transition structure* is a triple $\mathcal {M}^{ ts } = \langle M,<, ts \rangle$, where $\mathcal {M}=\langle M,<\rangle$ is a BT structure and $ts \subseteq \mathsf {dcts}(\mathcal {M})$ a non-empty set of transition sets such that for every moment $m \in M$, there is some $T \in ts$ such that ${\mathsf {H}}(T)\cap {\mathsf {H}}_m \ne \emptyset$.

#### **Definition 14**

(*Transition model*) A *transition model*${\mathfrak {M}}^{ ts } = \langle \mathcal {M}^{ ts },v_{\mathsf {t}}\rangle$ is a transition structure $\mathcal {M}^{ ts } = \langle M,<, ts \rangle$ together with a valuation function $v_{\mathsf {t}}: {{\mathsf {A}}}{{\mathsf {t}}}\times \{m/T\mid m\in M\text {, }T \in ts \text { and } {\mathsf {H}}(T)\cap {\mathsf {H}}_m \ne \emptyset \} \rightarrow \{0,1\}$.

The following semantic clauses extend the valuation $v_{\mathsf {t}}$ on the propositional variables $p \in {{\mathsf {A}}}{{\mathsf {t}}}$ in a transition model ${\mathfrak {M}}^{ ts } = \langle \mathcal {M}^{ ts },v_{\mathsf {t}}\rangle$ on a transition structure $\mathcal {M}^{ ts } = \langle M,<, ts \rangle$ to all sentences $\phi \in {\mathscr {L}}_{{\mathsf {t}}}$. As usual, we use ${\mathfrak {M}}^{ ts }\!,m/T \vDash _{{\mathsf {t}}}\phi$ in order to indicate that a sentence $\phi \in {\mathscr {L}}_{{\mathsf {t}}}$ is true in a transition model ${\mathfrak {M}}^{ ts }$ at a moment $m \in M$ with respect to a transition set $T \in ts$. The expressions ${\mathfrak {M}}^{ ts } \vDash _{{\mathsf {t}}}\phi$ for validity in a transition model, $\mathcal {M}^{ ts } \vDash _{{\mathsf {t}}}\phi$ for validity in a transition structure and $\vDash _{{\mathsf {t}}}\phi$ for general validity are defined in the obvious way.

($${{\mathsf {A}}}{{\mathsf {t}}}$$)
$${\mathfrak {M}}^{ ts }\!,m/T \vDash _{{\mathsf {t}}}p \text { iff } v_{\mathsf {t}}(p,m/T) = 1$$;
($$\lnot $$)
$${\mathfrak {M}}^{ ts }\!,m/T \vDash _{{\mathsf {t}}}\lnot \phi \text { iff } {\mathfrak {M}}^{ ts }\!,m/T \not \vDash _{{\mathsf {t}}}\phi $$;
($$\wedge $$)
$${\mathfrak {M}}^{ ts }\!,m/T \vDash _{{\mathsf {t}}}\phi \wedge \psi \text { iff } {\mathfrak {M}}^{ ts }\!,m/T \vDash _{{\mathsf {t}}}\phi \text { and } {\mathfrak {M}}^{ ts }\!,m/T \vDash _{{\mathsf {t}}}\psi $$;
($${\mathsf {F}}$$)
$${\mathfrak {M}}^{ ts }\!,m/T \vDash _{{\mathsf {t}}}{\mathsf {F}}\phi \text { iff for all }h \in {\mathsf {H}}(T)\cap {\mathsf {H}}_m, \text { there is some } m'\in h \text { s.t.}~m'>m \text { and }{\mathfrak {M}}^{ ts }\!,m'/T \vDash _{{\mathsf {t}}}\phi $$;
($${\mathsf {P}}$$)
$${\mathfrak {M}}^{ ts }\!,m/T \vDash _{{\mathsf {t}}}{\mathsf {P}}\phi \text { iff there is some } m'<m \text { s.t. } {\mathfrak {M}}^{ ts }\!,m'/T \vDash _{{\mathsf {t}}}\phi $$;
($$\Box $$)
$${\mathfrak {M}}^{ ts }\!,m/T \vDash _{{\mathsf {t}}}\Box \phi \text { iff for all } T' \in ts \text { s.t. } {\mathsf {H}}(T')\cap {\mathsf {H}}_m \ne \emptyset \text {, } {\mathfrak {M}}^{ ts }\!,m/T' \vDash _{{\mathsf {t}}}\phi $$;
($${\mathsf {S}}$$)
$${\mathfrak {M}}^{ ts }\!,m/T \vDash _{{\mathsf {t}}}{\mathsf {S}}\phi \text { iff for all } T' \in ts \text { s.t. } T' \supseteq T \text { and } {\mathsf {H}}(T')\cap {\mathsf {H}}_m \ne \emptyset , {\mathfrak {M}}^{ ts }\!, m/T' \vDash _{{\mathsf {t}}}\phi $$.

In a transition model ${\mathfrak {M}}^{ ts } = \langle \mathcal {M}^{ ts },v_{\mathsf {t}}\rangle$, the inevitability operator $\Box$ and the stability operator ${\mathsf {S}}$, as well as their duals $\Diamond := \lnot \Box \lnot$ and , quantify over transition sets in $ts$ only. The semantic clauses for the future operator ${\mathsf {F}}$ and the past operator ${\mathsf {P}}$ remain intact, as they do not involve a shift of the transition parameter. In the case of the future operator, we make use of the formulation in which the quantification over future possibilities is spelled out in terms of the set of histories admitted by the given transition set—rather than in terms of its possible future extensions. In Sect. 3.2, we focused on models on transition structures $\mathcal {M}^{\mathsf {dcts}(\mathcal {M})} = \langle M,<,\mathsf {dcts}(\mathcal {M})\rangle$, and the semantic clauses were formulated for only that class of models. Transition models on a structure $\mathcal {M}^{\mathsf {dcts}(\mathcal {M})}$ make use of the entire range of consistent, downward closed sets of transitions the BT structure $\mathcal {M}$ has to offer, while all other transition models rest upon limited resources only. With the general notion of a transition model at our disposal, let us now consider how Peirceanism and Ockhamism can be captured within the transition approach.

### Generalizing Peirceanism

Let us have a look at the Peircean account first. On the Peircean account, the semantic evaluation is relativized to a moment parameter only: sentences $\phi \in {\mathscr {L}}_{{\mathsf {p}}}$ are assigned truth values in a Peircean model ${\mathfrak {M}}= \langle \mathcal {M},v_{\mathsf {p}}\rangle$ on a BT structure $\mathcal {M}=\langle M,<\rangle$ relative to a moment $m \in M$. The clause for the future operator $\mathsf {F_p}$ universally quantifies over all histories containing the moment of evaluation, demanding a future witness in every single one of them. We can capture Peirceanism with its sole dependence on the moment of evaluation *m* in the transition framework by restricting the semantic evaluation on a BT structure $\mathcal {M}=\langle M,<\rangle$ to the empty transition set $\mathsf {\emptyset _{Tr}}$. In other words, we consider models on a transition structure $\mathcal {M}^{\{\mathsf {\emptyset _{Tr}}\}} = \langle M,<,\{\mathsf {\emptyset _{Tr}}\}\rangle$. Due to the restriction to the empty transition set $\mathsf {\emptyset _{Tr}}$, the possible indices of evaluation are restricted to pairs $m/\mathsf {\emptyset _{Tr}}$ consisting of a moment $m \in M$ and the empty transition set $\mathsf {\emptyset _{Tr}}$, for which it holds that ${\mathsf {H}}(\mathsf {\emptyset _{Tr}}) \cap {\mathsf {H}}_m = {\mathsf {H}}_m$, since ${\mathsf {H}}(\mathsf {\emptyset _{Tr}}) = \mathsf {hist}(\mathcal {M})$. At any moment of evaluation $m \in M$, all histories containing *m* are admitted by the empty transition set $\mathsf {\emptyset _{Tr}}$, which allows us to mimic the Peircean future operator. We call a transition structure $\mathcal {M}^{\{\mathsf {\emptyset _{Tr}}\}} = \langle M,<,\{\mathsf {\emptyset _{Tr}}\}\rangle$ a *Peircean transition structure* and a model ${\mathfrak {M}}^{\{\mathsf {\emptyset _{Tr}}\}} = \langle \mathcal {M}^{\{\mathsf {\emptyset _{Tr}}\}},v_{\mathsf {t}}\rangle$ on such a structure a *Peircean transition model*.

#### **Definition 15**

(*Peircean transition structure and model*) A transition structure $\mathcal {M}^{ ts } = \langle M,<, ts \rangle$ is called a *Peircean transition structure* iff $ts = \{\mathsf {\emptyset _{Tr}}\}$. A transition model ${\mathfrak {M}}^{\{\mathsf {\emptyset _{Tr}}\}} = \langle \mathcal {M}^{\{\mathsf {\emptyset _{Tr}}\}},v_{\mathsf {t}}\rangle$ on a Peircean transition structure $\mathcal {M}^{\{\mathsf {\emptyset _{Tr}}\}}$ is called a *Peircean transition model*.

There is a natural one-to-one correspondence between Peircean transition structures $\mathcal {M}^{\{\mathsf {\emptyset _{Tr}}\}} = \langle M,<,\{\mathsf {\emptyset _{Tr}}\}\rangle$ and BT structures $\mathcal {M}=\langle M,<\rangle$. We show that for every Peircean transition model ${\mathfrak {M}}^{\{\mathsf {\emptyset _{Tr}}\}} = \langle \mathcal {M}^{\{\mathsf {\emptyset _{Tr}}\}}\!,v_{\mathsf {t}}\rangle$, there is a Peircean BT model ${\mathfrak {M}}= \langle \mathcal {M},v_{\mathsf {p}}\rangle$, and *vice versa*, such that a sentence $\phi \in {\mathscr {L}}_{{\mathsf {t}}}$ is true in ${\mathfrak {M}}^{\{\mathsf {\emptyset _{Tr}}\}}$ at a pair $m/\mathsf {\emptyset _{Tr}}$ according to the transition semantics if and only if its respective translation $\phi ^*\in {\mathscr {L}}_{{\mathsf {p}}}$ is true in ${\mathfrak {M}}$ at the moment *m* according to the Peircean semantics. For any sentence $\phi \in {\mathscr {L}}_{{\mathsf {t}}}$, its translation $\phi ^*$ into the Peircean language ${\mathscr {L}}_{{\mathsf {p}}}$ can be defined recursively, and every sentence of the Peircean language is a translation of some sentence in the transition language. As both ${\mathscr {L}}_{{\mathsf {t}}}$ and ${\mathscr {L}}_{{\mathsf {p}}}$ are extensions of the propositional language ${\mathscr {L}}$, suffice it to say that $({\mathsf {F}}\phi )^*= \mathsf {F_p}\phi ^*$, $({\mathsf {P}}\phi )^*= \mathsf {P_p}\phi ^*$, $(\Box \phi )^*= \phi ^*$ and $({\mathsf {S}}\phi )^*= \phi ^*$. Note that due to the restriction to a single set of transitions, viz. the empty transition set $\mathsf {\emptyset _{Tr}}$, the equivalences $\Box p \leftrightarrow p$ and ${\mathsf {S}}p \leftrightarrow p$ are valid in every Peircean transition structure, in symbols: $\vDash _{{\mathsf {t}}}^{\{\mathsf {\emptyset _{Tr}}\}}\!\Box p \leftrightarrow p$ and $\vDash _{{\mathsf {t}}}^{\{\mathsf {\emptyset _{Tr}}\}}\!{\mathsf {S}}p \leftrightarrow p$.

#### **Proposition 4**

Let $\mathcal {M}^{\{\mathsf {\emptyset _{Tr}}\}} = \langle M,<,\{\mathsf {\emptyset _{Tr}}\}\rangle$ be a Peircean transition structure. The mapping $\xi : \langle \mathcal {M}^{\{\mathsf {\emptyset _{Tr}}\}}\!,v_{\mathsf {t}}\rangle \mapsto \langle \mathcal {M},v_{\mathsf {p}}\rangle$ with $v_{\mathsf {t}}(p,m/\mathsf {\emptyset _{Tr}}) = v_{\mathsf {p}}(p,m)$ for all $p \in {{\mathsf {A}}}{{\mathsf {t}}}$ and $m\in M$ is a bijection between Peircean transition models on $\mathcal {M}^{\{\mathsf {\emptyset _{Tr}}\}}$ and Peircean BT models on $\mathcal {M}$ . The following holds: given a Peircean transition model ${\mathfrak {M}}^{\{\mathsf {\emptyset _{Tr}}\}} = \langle \mathcal {M}^{\{\mathsf {\emptyset _{Tr}}\}}\!,v_{\mathsf {t}}\rangle$, for every $\phi \in {\mathscr {L}}_{{\mathsf {t}}}$ and every $m \in M$:

$$\begin{aligned} {\mathfrak {M}}^{\{\mathsf {\emptyset _{Tr}}\}}\!,m/\mathsf {\emptyset _{Tr}}\vDash _{{\mathsf {t}}}\phi \quad \text { iff } \quad \xi ({\mathfrak {M}}^{\{\mathsf {\emptyset _{Tr}}\}}),m \vDash _{{\mathsf {p}}}\phi ^*. \end{aligned}$$

#### *Proof*

The proof runs by induction on the structure of a sentence $\phi \in {\mathscr {L}}_{{\mathsf {t}}}$. Given the correspondence $\xi$, the base clause is straightforward. The case for ${\mathsf {P}}$ is trivial, and the cases for $\Box$ and ${\mathsf {S}}$ dissolve due to the validity of the equivalences $\Box p \leftrightarrow p$ and ${\mathsf {S}}p \leftrightarrow p$. In the case of the future operator ${\mathsf {F}}$, the proof makes use of the fact that ${\mathsf {H}}(\mathsf {\emptyset _{Tr}}) = \mathsf {hist}(\mathcal {M})$ and hence ${\mathsf {H}}(\mathsf {\emptyset _{Tr}}) \cap {\mathsf {H}}_m = {\mathsf {H}}_m$. $\square$

From Proposition 4 it follows that a sentence $\phi \in {\mathscr {L}}_{{\mathsf {t}}}$ is valid in a Peircean transition structure $\mathcal {M}^{\{\mathsf {\emptyset _{Tr}}\}}$ if and only if its translation $\phi ^*$ is Peircean valid in the corresponding BT structure $\mathcal {M}$ . Since there is a natural one-to-one correspondence between Peircean transition structures and BT structures, this implies that a sentence $\phi \in {\mathscr {L}}_{{\mathsf {t}}}$ is valid with respect to the class of all Peircean transition structures if and only if its translation $\phi ^*$ is valid in the Peircean semantics.

#### **Corollary 1**

For every $$\phi \in {\mathscr {L}}_{{\mathsf {t}}}$$:

$$\begin{aligned} \vDash _{{\mathsf {t}}}^{\{\mathsf {\emptyset _{Tr}}\}}\!\phi \quad \text { iff } \quad \vDash _{{\mathsf {p}}}\phi ^*. \end{aligned}$$

#### *Proof*

Follows from Proposition 4. $\square$

By Corollary 1, Peircean validity is equivalent to validity with respect to the class of all Peircean transition structures as every sentence of the Peircean language ${\mathscr {L}}_{{\mathsf {p}}}$ is a translation of some sentence in the transition language ${\mathscr {L}}_{{\mathsf {t}}}$. We show that the class of all Peircean transition structures can be characterized in ${\mathscr {L}}_{{\mathsf {t}}}$, which then allows us to define Peircean validity in the transition semantics. More precisely, we prove that a transition structure $\mathcal {M}^{ ts }$ is a Peircean transition structure if and only if the equivalence $\Box p \leftrightarrow p$ is valid in $\mathcal {M}^{ ts }$.

#### **Proposition 5**

For $\mathcal {M}^{ ts } = \langle M,<, ts \rangle$ a transition structure:

$$\begin{aligned} ts = \{\emptyset _{{\mathsf {Tr}}}\} \quad \text { iff } \quad \mathcal {M}^{ ts } \vDash _{{\mathsf {t}}}\Box p \leftrightarrow p. \end{aligned}$$

#### *Proof*

“$\Rightarrow$”: Assume that $ts = \{\mathsf {\emptyset _{Tr}}\}$. As said, we have $\vDash _{{\mathsf {t}}}^{\{\mathsf {\emptyset _{Tr}}\}}\!\Box p \leftrightarrow p$, and hence $\mathcal {M}^{ ts } \vDash _{{\mathsf {t}}}\Box p \leftrightarrow p$.

“$\Leftarrow$”: Assume that $ts \ne \{\mathsf {\emptyset _{Tr}}\}$. Then there are $T,T' \in \mathsf {dcts}(\mathcal {M})$ s.t. $ts \supseteq \{T,T'\}$. For, assume that $ts = \{T\}$ for some $T \ne \mathsf {\emptyset _{Tr}}$. Then there is some $\langle m\!\rightarrowtail \!H\rangle \in T$, which implies that there is some $h \in {\mathsf {H}}_m\setminus H$ and some $m' \in h$ s.t. $H \cap {\mathsf {H}}_{m'} = \emptyset$. Since ${\mathsf {H}}(T)\subseteq H$, it follows that ${\mathsf {H}}(T)\cap {\mathsf {H}}_{m'} = \emptyset$. Hence, for $ts = \{T\} \ne \{\mathsf {\emptyset _{Tr}}\}$, $\mathcal {M}^{ ts }$ is not a transition structure according to Definition 13. Now take some moment $m \in M$ s.t. ${\mathsf {H}}(T)\cap {\mathsf {H}}_m \ne \emptyset$ and ${\mathsf {H}}(T')\cap {\mathsf {H}}_m \ne \emptyset$. Consider a model ${\mathfrak {M}}^{ ts } = \langle \mathcal {M}^{ ts },v_{\mathsf {t}}\rangle$ s.t. $v_{\mathsf {t}}(p,m/T) = 1$ and $v_{\mathsf {t}}(p,m/T') = 0$. We then have that ${\mathfrak {M}}^{ ts }\!,m/T \vDash _{{\mathsf {t}}}p$ but ${\mathfrak {M}}^{ ts }\!,m/T \not \vDash _{{\mathsf {t}}}\Box p$, since ${\mathfrak {M}}^{ ts }\!,m/T' \not \vDash _{{\mathsf {t}}}p$. Consequently, ${\mathfrak {M}}^{ ts }\!,m/T \not \vDash _{{\mathsf {t}}}\Box p \leftrightarrow p$, and hence $\mathcal {M}^{ ts } \not \vDash _{{\mathsf {t}}}\Box p \leftrightarrow p$. $\square$

As said, by Corollary 1, Peircean validity is equivalent to validity with respect to the class of all Peircean transition structures, and by the correspondence result established in Proposition 5, that class can be characterized by the equivalence $\Box p \leftrightarrow p$. Peircean validity is thus definable in the transition semantics: a sentence of the Peircean language is valid in the Peircean semantics if and only if its ${\mathscr {L}}_{{\mathsf {t}}}$-correspondent is valid in all transition structures in which $\Box p \leftrightarrow p$ is valid.

#### **Corollary 2**

For every $$\phi \in {\mathscr {L}}_{{\mathsf {t}}}$$:

$$\begin{aligned} (\text {for all } \mathcal {M}^{ ts } \text { s.t. } \mathcal {M}^{ ts } \vDash _{{\mathsf {t}}}\Box p \leftrightarrow p\text {, } \mathcal {M}^{ ts } \vDash _{{\mathsf {t}}}\phi ) \quad \text { iff } \quad \vDash _{{\mathsf {p}}}\phi ^*. \end{aligned}$$

#### *Proof*

Follows from Corollary 1 and Proposition 5. $\square$

### Generalizing Ockhamism

On the Ockhamist account, the semantic evaluation is relativized to a history as a second parameter of truth next to the moment parameter: sentences $\phi \in {\mathscr {L}}_{{\mathsf {t}}}$ are assigned truth values in an Ockhamist model ${\mathfrak {M}}= \langle \mathcal {M},v_{\mathsf {o}}\rangle$ on a BT structure $\mathcal {M}=\langle M,<\rangle$ relative to a moment-history pair *m* / *h*, where $m \in M$ and $h \in {\mathsf {H}}_m$. The clause for the future operator $\mathsf {F_o}$ simply shifts the moment of evaluation forward on the given history, just as in linear tense logic. Since, by Propositions 2 and 3, there is a one-to-one correspondence between histories and maximal consistent transition sets, we can capture Ockhamism with its dependence on a history parameter in the transition semantics by restricting the semantic evaluation to maximal consistent transition sets. For $\mathcal {M}=\langle M,<\rangle$ a BT structure, let $\mathsf {mcts}(\mathcal {M})\subseteq \mathsf {dcts}(\mathcal {M})$ be the *set of all maximal consistent transition sets in*$\mathcal {M}$ . Since for every history $h \in \mathsf {hist}(\mathcal {M})$, there is a maximal consistent transition set ${{\mathsf {T}}}{{\mathsf {r}}}(h)$, and since every maximal consistent transition set is identical to some such set ${{\mathsf {T}}}{{\mathsf {r}}}(h)$, we have that $\mathsf {mcts}(\mathcal {M})= \{{{\mathsf {T}}}{{\mathsf {r}}}(h)\in \mathsf {dcts}(\mathcal {M})\mid h \in \mathsf {hist}(\mathcal {M})\}$. We then consider models on a transition structure $\mathcal {M}^{\mathsf {mcts}(\mathcal {M})} = \langle M,<,\mathsf {mcts}(\mathcal {M})\rangle$. The restriction to maximal consistent transition sets ensures that the possible indices of evaluation are restricted to pairs $m/{{\mathsf {T}}}{{\mathsf {r}}}(h)$ consisting of a moment $m \in M$ and a maximal consistent set of transitions ${{\mathsf {T}}}{{\mathsf {r}}}(h)\in \mathsf {mcts}(\mathcal {M})$, for which it holds, by Proposition 3, that ${\mathsf {H}}({{\mathsf {T}}}{{\mathsf {r}}}(h)) = \{h\}$. At any index of evaluation $m/{{\mathsf {T}}}{{\mathsf {r}}}(h)$, exactly one history containing the moment *m* is admitted by the maximal consistent transition set ${{\mathsf {T}}}{{\mathsf {r}}}(h)$, viz. the corresponding history *h*, which allows us to mimic the Ockhamist future operator. Note that in a model on a transition structure $\mathcal {M}^{\mathsf {mcts}(\mathcal {M})} = \langle M,<,\mathsf {mcts}(\mathcal {M})\rangle$, the modal operators likewise quantify over maximal consistent transition sets only, which is in accordance with their interpretation in the Ockhamist semantics. We call a transition structure $\mathcal {M}^{\mathsf {mcts}(\mathcal {M})} = \langle M,<,\mathsf {mcts}(\mathcal {M})\rangle$ an *Ockhamist transition structure* and a model ${\mathfrak {M}}^{\mathsf {mcts}(\mathcal {M})} = \langle \mathcal {M}^{\mathsf {mcts}(\mathcal {M})},v_{\mathsf {t}}\rangle$ on such a structure an *Ockhamist transition model*.

#### **Definition 16**

(*Ockhamist transition structure and model*) A transition structure $\mathcal {M}^{ ts } = \langle M,<, ts \rangle$ is called an *Ockhamist transition structure* iff $ts = \mathsf {mcts}(\mathcal {M})$. A transition model ${\mathfrak {M}}^{\mathsf {mcts}(\mathcal {M})} = \langle \mathcal {M}^{\mathsf {mcts}(\mathcal {M})},v_{\mathsf {t}}\rangle$ on an Ockhamist transition structure $\mathcal {M}^{\mathsf {mcts}(\mathcal {M})}$ is called an *Ockhamist transition model*.

There is a natural one-to-one correspondence between Ockhamist transition structures $\mathcal {M}^{\mathsf {mcts}(\mathcal {M})} = \langle M,<,\mathsf {mcts}(\mathcal {M})\rangle$ and BT structures $\mathcal {M}=\langle M,<\rangle$. Just as in the Peircean case, we show that for every Ockhamist transition model ${\mathfrak {M}}^{\mathsf {mcts}(\mathcal {M})} = \langle \mathcal {M}^{\mathsf {mcts}(\mathcal {M})}\!,v_{\mathsf {t}}\rangle$, there is an Ockhamist BT model ${\mathfrak {M}}= \langle \mathcal {M},v_{\mathsf {o}}\rangle$, and *vice versa*, such that a sentence $\phi \in {\mathscr {L}}_{{\mathsf {t}}}$ is true in ${\mathfrak {M}}^{\mathsf {mcts}(\mathcal {M})}$ at a pair $m/{{\mathsf {T}}}{{\mathsf {r}}}(h)$ according to the transition semantics if and only if its respective translation $\phi ^*\in {\mathscr {L}}_{{\mathsf {o}}}$ is true in ${\mathfrak {M}}$ at the corresponding pair *m* / *h* according to the Ockhamist semantics. For any sentence $\phi \in {\mathscr {L}}_{{\mathsf {t}}}$, its translation $\phi ^*$ into the Ockhamist language ${\mathscr {L}}_{{\mathsf {o}}}$ can be defined recursively, and every sentence of the Ockhamist language is a translation of some sentence in the transition language. As both ${\mathscr {L}}_{{\mathsf {t}}}$ and ${\mathscr {L}}_{{\mathsf {o}}}$ are extensions of the propositional language ${\mathscr {L}}$, suffice it to say that $({\mathsf {F}}\phi )^*= \mathsf {F_o}\phi ^*$, $({\mathsf {P}}\phi )^*= \mathsf {P_o}\phi ^*$, $(\Box \phi )^*= \mathsf {\Box _o}\phi ^*$ and $({\mathsf {S}}\phi )^*= \phi ^*$. Note that due to the restriction to maximal consistent transition sets, which cannot be further extended, the equivalence ${\mathsf {S}}p \leftrightarrow p$ is valid in every Ockhamist transition structure, in symbols: $\vDash _{{\mathsf {t}}}^{\mathsf {mcts}}\!{\mathsf {S}}p \leftrightarrow p$.

#### **Proposition 6**

Let $\mathcal {M}^{\mathsf {mcts}(\mathcal {M})} = \langle M,<,\mathsf {mcts}(\mathcal {M})\rangle$ be an Ockhamist transition structure. The mapping $\xi : \langle \mathcal {M}^{\mathsf {mcts}(\mathcal {M})}\!,v_{\mathsf {t}}\rangle \mapsto \langle \mathcal {M},v_{\mathsf {o}}\rangle$ with $v_{\mathsf {t}}(p,m/{{\mathsf {T}}}{{\mathsf {r}}}(h)) = v_{\mathsf {o}}(p,m/h)$ for all $p \in {{\mathsf {A}}}{{\mathsf {t}}}$, $m\in M$ and $h \in {\mathsf {H}}_m$ is a bijection between Ockhamist transition models on $\mathcal {M}^{\mathsf {mcts}(\mathcal {M})}$ and Ockhamist BT models on $\mathcal {M}$ . The following holds: given an Ockhamist transition model ${\mathfrak {M}}^{\mathsf {mcts}(\mathcal {M})} = \langle \mathcal {M}^{\mathsf {mcts}(\mathcal {M})}\!,v_{\mathsf {t}}\rangle$, for every $\phi \in {\mathscr {L}}_{{\mathsf {t}}}$, every $m \in M$ and every $h \in {\mathsf {H}}_m$:

$$\begin{aligned} {\mathfrak {M}}^{\mathsf {mcts}(\mathcal {M})}\!,m/{{\mathsf {T}}}{{\mathsf {r}}}(h)\vDash _{{\mathsf {t}}}\phi \quad \text { iff } \quad \xi ({\mathfrak {M}}^{\mathsf {mcts}(\mathcal {M})}),m/h \vDash _{{\mathsf {o}}}\phi ^*. \end{aligned}$$

#### *Proof*

The proof runs by induction on the structure of a sentence $\phi \in {\mathscr {L}}_{{\mathsf {t}}}$. Given the correspondence $\xi$, the base clause is straightforward. The case for ${\mathsf {P}}$ is trivial, and the case for ${\mathsf {S}}$ dissolves due to the validity of the equivalence ${\mathsf {S}}p \leftrightarrow p$. In the case of the future operator ${\mathsf {F}}$ and the inevitability operator $\Box$, the proof makes use of the fact that $\mathsf {mcts}(\mathcal {M})= \{{{\mathsf {T}}}{{\mathsf {r}}}(h)\in \mathsf {dcts}(\mathcal {M})\mid h \in \mathsf {hist}(\mathcal {M})\}$ and ${\mathsf {H}}({{\mathsf {T}}}{{\mathsf {r}}}(h)) = \{h\}$ for all $h \in \mathsf {hist}(\mathcal {M})$. $\square$

Due to the one-to-one correspondence between Ockhamist transition models and Ockhamist BT models established in Proposition 6, validity with respect to the class of all Ockhamist transition structures coincides with Ockhamist validity: a sentence $\phi \in {\mathscr {L}}_{{\mathsf {t}}}$ is valid in every Ockhamist transition structure if and only if its translation $\phi ^*\in {\mathscr {L}}_{{\mathsf {o}}}$ is valid in the Ockhamist semantics.

#### **Corollary 3**

For every $$\phi \in {\mathscr {L}}_{{\mathsf {t}}}$$:

$$\begin{aligned} \vDash _{{\mathsf {t}}}^{\mathsf {mcts}}\!\phi \quad \text { iff } \quad \vDash _{{\mathsf {o}}}\phi ^*. \end{aligned}$$

#### *Proof*

Follows from Proposition 6. $\square$

Since every sentence of the Ockhamist language ${\mathscr {L}}_{{\mathsf {o}}}$ is a translation of some sentence in the transition language ${\mathscr {L}}_{{\mathsf {t}}}$, by Corollary 3, Ockhamist validity is equivalent to validity with respect to the class of all Ockhamist transition structures.

We finally show that the class of all Ockhamist transition structures can be characterized in ${\mathscr {L}}_{{\mathsf {t}}}$, which allows us to define Ockhamist validity in the transition semantics. We proceed in two steps: we first prove that the class of all transition structures $\mathcal {M}^{ ts }$ in which $ts$ comprises maximal consistent transition sets only, i.e., $ts \subseteq \mathsf {mcts}(\mathcal {M})$, is definable in ${\mathscr {L}}_{{\mathsf {t}}}$. We then single out from that class those transition structures $\mathcal {M}^{ ts }$ for which $ts = \mathsf {mcts}(\mathcal {M})$.

We now show in a first step that the class of all transition structures $\mathcal {M}^{ ts }$ in which $ts$ does not contain any transition set that is not maximal consistent can be characterized in ${\mathscr {L}}_{{\mathsf {t}}}$. In particular, we prove that for all transition structures $\mathcal {M}^{ ts }$ it holds that $ts \subseteq \mathsf {mcts}(\mathcal {M})$ if and only if the disjunction ${\mathsf {F}}p \vee {\mathsf {F}}\lnot p$ is valid in $\mathcal {M}^{ ts }$.

#### **Proposition 7**

For $\mathcal {M}^{ ts } = \langle M,<, ts \rangle$ a transition structure:

$$\begin{aligned} ts \subseteq \mathsf {mcts}(\mathcal {M})\quad \text { iff } \quad \mathcal {M}^{ ts } \vDash _{{\mathsf {t}}}{\mathsf {F}}p \vee {\mathsf {F}}\lnot p. \end{aligned}$$

#### *Proof*

“$\Rightarrow$”: Let $\mathcal {M}^{ ts } = \langle M,<, ts \rangle$ be a transition structure with $ts \subseteq \mathsf {mcts}(\mathcal {M})$. Let ${\mathfrak {M}}^{ ts }$ be a model on $\mathcal {M}^{ ts }$, and let $m\in M$ and ${{\mathsf {T}}}{{\mathsf {r}}}(h)\in ts$ s.t. ${\mathsf {H}}({{\mathsf {T}}}{{\mathsf {r}}}(h)) \cap {\mathsf {H}}_m \ne \emptyset$. Assume that ${\mathfrak {M}}^{ ts }\!,m/{{\mathsf {T}}}{{\mathsf {r}}}(h)\not \vDash _{{\mathsf {t}}}{\mathsf {F}}p$. By the semantic clause for ${\mathsf {F}}$, it follows that for all $m' \in M$ s.t. $m'\in h$ and $m'>m$, ${\mathfrak {M}}^{ ts }\!,m'/{{\mathsf {T}}}{{\mathsf {r}}}(h)\not \vDash _{{\mathsf {t}}}p$, which again implies that ${\mathfrak {M}}^{ ts }\!,m/{{\mathsf {T}}}{{\mathsf {r}}}(h)\vDash _{{\mathsf {t}}}{\mathsf {F}}\lnot p$ since, by condition (BT3) of Definition 1, there is no last moment. Hence, ${\mathfrak {M}}^{ ts }\!,m/{{\mathsf {T}}}{{\mathsf {r}}}(h)\vDash _{{\mathsf {t}}}{\mathsf {F}}p \vee {\mathsf {F}}\lnot p$. As ${\mathfrak {M}}^{ ts }$, *m* and ${{\mathsf {T}}}{{\mathsf {r}}}(h)$ were arbitrarily chosen, it follows that $\mathcal {M}^{ ts } \vDash _{{\mathsf {t}}}{\mathsf {F}}p \vee {\mathsf {F}}\lnot p$.

“$\Leftarrow$”: Assume that $ts \not \subseteq \mathsf {mcts}(\mathcal {M})$. Then there is some $T \in ts$ s.t. *T* is not maximal consistent, i.e., there is some transition $\langle m\!\rightarrowtail \!H\rangle \in \mathsf {trans}(\mathcal {M})$ s.t. $\langle m\!\rightarrowtail \!H\rangle \notin T$ and ${\mathsf {H}}(T \cup \{\langle m\!\rightarrowtail \!H\rangle \}) \ne \emptyset$. This implies that ${\mathsf {H}}_m \subseteq {\mathsf {H}}(T)$ and that there are histories $h,h' \in {\mathsf {H}}_m$ s.t. $h \perp _m h'$. Consider some transition model ${\mathfrak {M}}^{ ts } = \langle \mathcal {M}^{ ts },v_{\mathsf {t}}\rangle$ s.t. $v_{\mathsf {t}}(p,m'/T) = 1$ if $m'>m$ and $m' \in h$, and $v_{\mathsf {t}}(p,m'/T) = 0$ if $m'>m$ and $m' \in h'$. We then have that ${\mathfrak {M}}^{ ts }\!,m/T \not \vDash _{{\mathsf {t}}}{\mathsf {F}}p$ and ${\mathfrak {M}}^{ ts }\!,m/T \not \vDash _{{\mathsf {t}}}{\mathsf {F}}\lnot p$. Consequently, ${\mathfrak {M}}^{ ts }\!,m/T \not \vDash _{{\mathsf {t}}}{\mathsf {F}}p \vee {\mathsf {F}}\lnot p$, and hence $\mathcal {M}^{ ts } \not \vDash _{{\mathsf {t}}}{\mathsf {F}}p \vee {\mathsf {F}}\lnot p$. $\square$

Now consider the class of all transition structures $\mathcal {M}^{ ts }$ with $ts \subseteq \mathsf {mcts}(\mathcal {M})$. If $ts = \mathsf {mcts}(\mathcal {M})= \{{{\mathsf {T}}}{{\mathsf {r}}}(h)\in \mathsf {dcts}(\mathcal {M})\mid h \in \mathsf {hist}(\mathcal {M})\}$, then $\mathcal {M}^{ ts }$ is an Ockhamist transition structure. However, it is also possible that $ts \subsetneq \mathsf {mcts}(\mathcal {M})$ although we have $\bigcup _{{{\mathsf {T}}}{{\mathsf {r}}}(h)\in ts }h = M$ as required by Definition 13, and in that case $\mathcal {M}^{ ts }$ is not an Ockhamist transition structure.^13^ Validity with respect to the class of all transition structures $\mathcal {M}^{ ts }$ with $ts \subseteq \mathsf {mcts}(\mathcal {M})$ coincides with validity with respect to the class of all so-called *bundled trees* rather than with Ockhamist validity. A bundled tree $\mathcal {B}= \langle M,<,B\rangle$ is a BT structure $\mathcal {M}=\langle M,<\rangle$ together with a non-empty set *B* of histories such that for every moment $m \in M$, there is a history $h \in B$ such that $m \in h$. A bundled tree is the Ockhamist equivalent to a transition structure: a bundled tree includes a set of primitive histories that spans the entire set of moments, while a transition structure includes a set of primitive consistent, downward closed transition sets that covers the whole structure.

#### **Definition 17**

(*Bundled tree*) A *bundled tree* is a triple $\mathcal {B}= \langle M,<,B\rangle$, where $\mathcal {M}=\langle M,<\rangle$ is a BT structure and $B \subseteq \mathsf {hist}(\mathcal {M})$ a non-empty set of histories such that for every moment $m \in M$, there is some $h \in B$ such that $m \in h$. A bundled tree $\mathcal {B}= \langle M,<,B\rangle$ is said to be *complete* iff $B = \mathsf {hist}(\mathcal {M})$.

In a model ${\mathfrak {B}}= \langle \mathcal {B},v_{\mathsf {o}}\rangle$ on a bundled tree $\mathcal {B}= \langle M,<,B\rangle$, sentences $\phi \in {\mathscr {L}}_{{\mathsf {o}}}$ are evaluated at moment-history pairs *m* / *T* with $m\in M$ and $h \in B$ such that $m\in h$. That is, only histories $h \in B$ are taken into account in the semantic evaluation, and the domain of quantification of the inevitability operator $\mathsf {\Box _o}$ is likewise restricted to the set *B*. We use ${\mathfrak {B}},m/h \vDash _{{\mathsf {o}}}\phi$ in order to indicate that a sentence $\phi \in {\mathscr {L}}_{{\mathsf {o}}}$ is true at a pair *m* / *h* in a model ${\mathfrak {B}}= \langle \mathcal {B},v_{\mathsf {o}}\rangle$ on a bundled tree $\mathcal {B}= \langle M,<,B\rangle$ with $v_{\mathsf {o}}: {{\mathsf {A}}}{{\mathsf {t}}}\times \{m/T\mid m \in M \text { and } h \in B \text { s.t. } m\in h\} \rightarrow \{0,1\}$ according to the (restricted) Ockhamist semantics. The notation extends to ${\mathfrak {B}}\vDash _{{\mathsf {o}}}\phi$, $\mathcal {B}\vDash _{{\mathsf {o}}}\phi$ and $\vDash _{{\mathsf {o}}}^{\mathsf {b}} \phi$ in the obvious way.

There is a natural one-to-one correspondence between transition structures $\mathcal {M}^{ ts } = \langle M,<, ts \rangle$ with $ts \subseteq \mathsf {mcts}(\mathcal {M})$ and bundled trees $\mathcal {B}= \langle M,<,B\rangle$ with $B = \{h \in \mathsf {hist}(\mathcal {M})\mid {{\mathsf {T}}}{{\mathsf {r}}}(h)\in ts \}$. Note that the structure $\mathcal {B}= \langle M,<,B\rangle$ with $B = \{h\in \mathsf {hist}(\mathcal {M})\mid {{\mathsf {T}}}{{\mathsf {r}}}(h)\in ts \}$ is a bundled tree according to Definition 17 if and only if the structure $\mathcal {M}^{ ts } = \langle M,<, ts \rangle$ with $ts \subseteq \mathsf {mcts}(\mathcal {M})$ is a transition structure according to Definition 13: we have that for every $m \in M$, there is some history $h \in B$ such that $m \in h$ iff for every $m\in M$, there is some maximal consistent transition set ${{\mathsf {T}}}{{\mathsf {r}}}(h)\in ts$ such that ${\mathsf {H}}({{\mathsf {T}}}{{\mathsf {r}}}(h)) \cap {\mathsf {H}}_m \ne \emptyset$. Obviously, the bundled tree $\mathcal {B}= \langle M,<,B\rangle$ with $B = \{h\in \mathsf {hist}(\mathcal {M})\mid {{\mathsf {T}}}{{\mathsf {r}}}(h)\in ts \}$ is complete if and only if $ts = \mathsf {mcts}(\mathcal {M})$. We show that a transition structure $\mathcal {M}^{ ts } = \langle M,<, ts \rangle$ with $ts \subseteq \mathsf {mcts}(\mathcal {M})$ validates a sentence $\phi \in {\mathscr {L}}_{{\mathsf {t}}}$ if and only if the corresponding bundled tree $\mathcal {B}= \langle M,<,B\rangle$ with $B = \{h\in \mathsf {hist}(\mathcal {M})\mid {{\mathsf {T}}}{{\mathsf {r}}}(h)\in ts \}$ validates its Ockhamist translation $\phi ^*\in {\mathscr {L}}_{{\mathsf {o}}}$.

#### **Proposition 8**

Let $\mathcal {M}^{ ts } = \langle M,<, ts \rangle$ be a transition structure with $ts \subseteq \mathsf {mcts}(\mathcal {M})$, and let $\mathcal {B}= \langle M,<,B\rangle$ be the bundled tree with $B = \{h \in \mathsf {hist}(\mathcal {M})\mid {{\mathsf {T}}}{{\mathsf {r}}}(h)\in ts \}$. The mapping $\xi : \langle \mathcal {M}^{ ts }\!,v_{\mathsf {t}}\rangle \mapsto \langle \mathcal {B},v_{\mathsf {o}}\rangle$ with $v_{\mathsf {t}}(p,m/{{\mathsf {T}}}{{\mathsf {r}}}(h)) = v_{\mathsf {o}}(p,m/h)$ for all $p \in {{\mathsf {A}}}{{\mathsf {t}}}$, $m\in M$ and $h \in B$ s.t. $m\in h$ is a bijection between transition models on $\mathcal {M}^{ ts }$ and bundled tree models on $\mathcal {B}$ . The following holds: given a transition model ${\mathfrak {M}}^{ ts } = \langle \mathcal {M}^{ ts }\!,v_{\mathsf {t}}\rangle$, for every $\phi \in {\mathscr {L}}_{{\mathsf {t}}}$, every $m \in M$ and every $h \in B$ s.t. $m\in h$:

$$\begin{aligned} {\mathfrak {M}}^{ ts }\!,m/{{\mathsf {T}}}{{\mathsf {r}}}(h)\vDash _{{\mathsf {t}}}\phi \quad \text { iff } \quad \xi ({\mathfrak {M}}^{ ts }),m/h \vDash _{{\mathsf {o}}}\phi ^*. \end{aligned}$$

#### *Proof*

The proof runs by induction on the structure of a sentence $\phi \in {\mathscr {L}}_{{\mathsf {t}}}$. Given the correspondence $\xi$, the base clause is straightforward. The case for ${\mathsf {P}}$ is trivial, and the case for ${\mathsf {S}}$ dissolves due to the validity of the equivalence ${\mathsf {S}}p \leftrightarrow p$. In the case of the future operator ${\mathsf {F}}$ and the inevitability operator $\Box$, the proof makes use of the fact that $B = \{h \in \mathsf {hist}(\mathcal {M})\mid {{\mathsf {T}}}{{\mathsf {r}}}(h)\in ts \}$ and ${\mathsf {H}}({{\mathsf {T}}}{{\mathsf {r}}}(h)) = \{h\}$ for all $h \in B$. $\square$

From Proposition 8 it follows that validity with respect to the class of all transition structures $\mathcal {M}^{ ts }$ with $ts \subseteq \mathsf {mcts}(\mathcal {M})$ is equivalent to validity with respect to the class of all bundled trees, as there is a natural one-to-one correspondence between the elements of those two classes. Since by the correspondence result established in Proposition 7 the former class can be characterized by the disjunction ${\mathsf {F}}p \vee {\mathsf {F}}\lnot p$, bundled tree validity is definable in the transition semantics.

#### **Corollary 4**

For every $$\phi \in {\mathscr {L}}_{{\mathsf {t}}}$$:

$$\begin{aligned} (\text {for all } \mathcal {M}^{ ts } \text { s.t. } \mathcal {M}^{ ts } \vDash _{{\mathsf {t}}}{\mathsf {F}}p \vee {\mathsf {F}}\lnot p\text {, } \mathcal {M}^{ ts } \vDash _{{\mathsf {t}}}\phi ) \quad \text { iff } \quad \vDash _{{\mathsf {o}}}^{\mathsf {b}} \phi ^*. \end{aligned}$$

#### *Proof*

Follows from Propositions 7 and 8. $\square$

As is well known, however, validity with respect to the class of all bundled trees is distinct from Ockhamist validity.^14^ Ockhamist validity is validity with respect to the class of all *complete* bundled trees. While $\vDash _{{\mathsf {o}}}^{\mathsf {b}}$ implies $\vDash _{{\mathsf {o}}}$, the opposite direction does not hold. In order to define Ockhamist validity in the transition semantics, we need to single out from the class of all transition structures $\mathcal {M}^{ ts }$ with $ts \subseteq \mathsf {mcts}(\mathcal {M})$ those for which $\mathsf {mcts}(\mathcal {M})\subseteq ts$ holds as well. In other words, we need to define within the class of all transition structures $\mathcal {M}^{ ts }$ such that $\mathcal {M}^{ ts } \vDash _{{\mathsf {t}}}{\mathsf {F}}p \vee {\mathsf {F}}\lnot p$, the class of those transition structures $\mathcal {M}^{ ts }$ for which $ts = \mathsf {mcts}(\mathcal {M})$. By Proposition 8, this amounts to characterizing the class of complete bundled trees within the class of bundled trees.

In Zanardo et al. (1999) it is shown that although in general it is not possible to define the class of complete bundled trees within the class of bundled trees, the class of complete bundled trees can be defined within the class of all bundled trees that are such that the intersection of any two histories contains a greatest element. Condition (BT2) of Definition 1 guarantees that this is here the case. It is the requirement that any two moments have a greatest common lower bound, that we have inserted in order to be able to define branching points, which figure as the initials of our transitions, in a perspicuous way, that allows us to apply the correspondence result provided in Zanardo et al. (1999). On the basis of that result, we can characterize the class of Ockhamist transition structures within the class of all transition structures $\mathcal {M}^{ ts }$ with $ts \subseteq \mathsf {mcts}(\mathcal {M})$ and hence define Ockhamist validity with respect to our BT structures in the transition semantics. Let $\delta := \Box {\mathsf {F}}\Box {\mathsf {G}}p \wedge \Diamond {\mathsf {F}}\lnot p \rightarrow \Diamond {\mathsf {F}}(\Box {\mathsf {G}}p \wedge {\mathsf {H}}(\lnot p \rightarrow {\mathsf {F}}\lnot p))$ with ${\mathsf {G}}= \lnot {\mathsf {F}}\lnot$ and ${\mathsf {H}}= \lnot {\mathsf {P}}\lnot$ be the ${\mathscr {L}}_{{\mathsf {t}}}$- correspondent of the characterizing formula provided in Zanardo et al. (1999).

#### **Corollary 5**

For every $$\phi \in {\mathscr {L}}_{{\mathsf {t}}}$$:

$$\begin{aligned} (\text {for all } \mathcal {M}^{ ts } \text { s.t. } \mathcal {M}^{ ts } \vDash _{{\mathsf {t}}}{\mathsf {F}}p \vee {\mathsf {F}}\lnot p \text { and } \mathcal {M}^{ ts } \vDash _{{\mathsf {t}}}\delta \text {, } \mathcal {M}^{ ts } \vDash _{{\mathsf {t}}}\phi ) \quad \text { iff } \quad \vDash _{{\mathsf {o}}}\phi ^*. \end{aligned}$$

#### *Proof*

“$\Rightarrow$”: Since $\mathsf {F_o}\,p \vee \mathsf {F_o}\lnot p$ and $\delta ^*$ are Ockhamist validities, by Corollary 3, we have that $\vDash _{{\mathsf {t}}}^{\mathsf {mcts}}\!{\mathsf {F}}p \vee {\mathsf {F}}\lnot p$ and $\vDash _{{\mathsf {t}}}^{\mathsf {mcts}}\!\delta$. From the assumption it thus follows that $\vDash _{{\mathsf {t}}}^{\mathsf {mcts}}\!\phi$, which implies by Corollary 3 that $\vDash _{{\mathsf {o}}}\phi ^*$.

“$\Leftarrow$”: Assume that $\vDash _{{\mathsf {o}}}\phi ^*$. By Corollary 3, it follows that $\vDash _{{\mathsf {t}}}^{\mathsf {mcts}}\!\phi$. Let $\mathcal {M}^{ ts } = \langle M,<, ts \rangle$ be a transition structure s.t. $\mathcal {M}^{ ts } \vDash _{{\mathsf {t}}}{\mathsf {F}}p \vee {\mathsf {F}}\lnot p$ and $\mathcal {M}^{ ts } \vDash _{{\mathsf {t}}}\delta$. By Proposition 7, $\mathcal {M}^{ ts } \vDash _{{\mathsf {t}}}{\mathsf {F}}p \vee {\mathsf {F}}\lnot p$ implies $ts \subseteq \mathsf {mcts}(\mathcal {M})$. Let $\mathcal {B}= \langle M,<,B\rangle$ be the bundled tree with $B = \{h\in \mathsf {hist}(\mathcal {M})\mid {{\mathsf {T}}}{{\mathsf {r}}}(h)\in ts \}$. By Proposition 8 it follows that $\mathcal {B}\vDash _{{\mathsf {o}}}\delta ^*$, which by the result provided in Zanardo et al. (1999) implies that $\mathcal {B}$ is complete, i.e., $B = \mathsf {hist}(\mathcal {M})$, and hence we also have $ts = \mathsf {mcts}(\mathcal {M})$. Since $\vDash _{{\mathsf {t}}}^{\mathsf {mcts}}\!\phi$, it follows that $\mathcal {M}^{ ts } \vDash _{{\mathsf {t}}}\phi$. $\square$

### Extending Extant Approaches

The results provided in Sects. 4.2 and 4.3 above demonstrate that both Peirceanism and Ockhamism can be captured in the transition framework. With the notion of a transition structure at our disposal, we can show that the transition semantics unifies the Peircean and the Ockhamist account and generalizes both of them. Both Peirceanism and Ockhamism turn out as limiting cases of the transition approach that are obtained by placing suitable restrictions on the transition sets employed in the semantic evaluation and, thus, by restricting the class of transition structures. Peircean validity coincides with validity with respect to the empty transition set, i.e. with respect to the class of all Peircean transition structures. Ockhamist validity, on the other hand, is equivalent to validity with respect to all maximal consistent transition sets, i.e. with respect to the class of all Ockhamist transition structures. Both Peircean and Ockhamist validity are definable in the transition semantics. Whereas the transition semantics in its most general form, as spelled out in Sect. 3.2, exploits the entire range of consistent, downward closed transition sets a BT structure has to offer, Peirceanism and Ockhamism each rest upon only a proper subset thereof.

The transition semantics does, however, not only generalize both Peirceanism and Ockhamism but also exceeds both accounts with respect to expressive strength. On the Peircean account, where the semantic evaluation depends solely on a moment parameter, inevitability and truth coincide: a sentence cannot be true without its truth being inevitable. The equivalence $\Box p \leftrightarrow p$ and hence also ${\mathsf {S}}p \leftrightarrow p$ are valid in every Peircean transition structure. On the Ockhamist account, where the semantic evaluation at a moment is in addition relativized to a complete possible course of events, inevitability and truth come apart, but stability and truth still coincide: a sentence cannot be true without being stably-true. In every Ockhamist transition structure, the equivalence ${\mathsf {S}}p \leftrightarrow p$ is valid. Neither on the Peircean nor on the Ockhamist account is an instance of  satisfiable. Since, compared to the transition semantics, Peirceanism and Ockhamism build on limited resources only, the stability operator loses its power: neither Peirceanism nor Ockhamism allow for quantification over proper future extensions. On the Peircean account, we are provided with just a single transition set, and the maximal consistent transition sets that the Ockhamist account rests on cannot be further extended. Building on the entire range of consistent, downward closed transition sets, the transition semantics allows for a more fine-grained picture of the interrelation of modality and time and gains expressive means that are not available on either of those accounts.

Zanardo (1998) provides a branching time semantics, which aims at retrieving the resources provided by a BT structure and generalizes both Peirceanism and Ockhamism, just as the transition approach does. On Zanardo’s account, sentences are evaluated on so-called *I*-*trees*, which are BT structures $\mathcal {M}=\langle M,<\rangle$ together with an indistinguishability function *I* that assigns to each moment $m \in M$ a partition of the set ${\mathsf {H}}_m$ of histories containing that moment. In a model on an *I*-tree, the semantic evaluation is relativized to pairs consisting of a moment $m \in M$ and an equivalence class modulo indistinguishability $[h]_m^I$ at that very moment. In case the indistinguishability relation is the relation of undividedness, evaluating a sentence at an index $m/[h]_m^I$ amounts to evaluating the sentence at the moment *m* with respect to the downward completion of the singleton of the transition $\langle m\!\rightarrowtail \![h]_m\rangle$ in the transition semantics.^15^ Just as the transition semantics, the *I*-tree framework thus allows for the relativization of the semantic evaluation to incomplete possible courses of events. However, unlike in the transition semantics, in the *I*-tree framework, indistinguishability classes cannot be shifted independently of the moment of evaluation: a sentence cannot be evaluated at a moment with respect to an indistinguishability class at another moment. Quantifying over extensions of a given transition set while keeping the moment of evaluation fixed, as in the case of the stability operator, is therefore not possible in the *I*-tree framework. It is not only by employing a second parameter of truth that can capture an incomplete possible course of events, but also by allowing that second parameter to vary independently of the moment parameter that the transition semantics gains its expressive power. Due to that combination the transition allows for a perspicuous treatment of future contingents.

A future contingent is a sentence about the future used in a context in which its truth value is not yet settled. Whether the sentence is true or false in that context depends on how the future unfolds. It is true in one possible future but false in another, with nothing yet deciding between those possibilities. On the Peircean account, where truth coincides with inevitability, all future contingents are rendered false at the moment of the context of use. The recurring problem of Ockhamism is that the history parameter cannot be initialized in a context of use: while the context of use provides a unique moment of use, it cannot single out one of the histories passing through that moment.^16^ Due to the mismatch between the parameters employed in the recursive semantics and the parameters provided by a context of use, Ockhamism is in need of a postsemantics that links the recursive semantic machinery to a context of use.^17^ There are two popular postsemantic accounts for Ockhamism: supervaluation, as put forth in Thomason (1970), and assessment-sensitivity, as provided in MacFarlane (2003, 2014). In MacFarlane’s assessment-sensitive postsemantics, stand-alone sentences are evaluated at a pair consisting of a moment of use $m_u$ and a later moment of assessment $m_a$. The moment of assessment provides a second perspective from which the truth of a future contingent at the moment of use can be retrospectively assessed. A sentence is said to be postsemantically-true if and only if it is true at the moment of use $m_u$ with respect to all histories containing the moment of assessment $m_a$ according to the Ockhamist semantics.^18^ Supervaluation can be viewed as a special case of the assessment-sensitive postsemantics, viz. the case in which the moment of assessment $m_a$ is identical to the moment of use $m_u$.

Since in the transition semantics truth values are assigned at a moment with respect to a consistent, downward closed set of transitions rather than with respect to an entire history, it is possible to extract an initial value for the second parameter of truth from a context of use. A sentence can be evaluated at a moment with respect to its past: the context of use specifies the moment of use $m_u$, which in turn determines the set ${{\mathsf {T}}}{{\mathsf {r}}}(m_u)$ of transitions preceding that moment. Moreover, the transition semantics allows us to express that a future contingent is contingent with respect to the parameters provided by the context of use, and the stability operator provides a means to specify how and how far the future has to unfold for its truth value at the moment of use to become settled. Future contingents are neither stably-true nor stably-false at the moment of use $m_u$ with respect to the transition set ${{\mathsf {T}}}{{\mathsf {r}}}(m_u)$ that captures the past course of events up to that moment. If a future contingent is stably-true relative to an index $m_u/{{\mathsf {T}}}{{\mathsf {r}}}(m_a)$, the moment $m_a$ constitutes a suitable moment of assessment in MacFarlane’s sense. The transition semantics provides a second perspective from which the truth value of a future contingent at the moment of use can be retrospectively assessed within the semantics itself. No postsemantics and no additional parameters are needed. By exploiting the entire range of consistent, downward closed sets of transitions a BT structure has to offer and allowing the stability operator to quantify over extensions of a given transition set, the transition semantics enables us to capture the behavior of the truth value of a future contingent in the course of time.

## Conclusion

In the transition semantics, the parameters of truth are provided by a moment and a consistent, downward closed set of transitions, which can represent a partial or complete possible course of events. In addition to temporal and modal operators, the transition language is equipped with a stability operator, which is interpreted as a universal quantifier over the possible extensions of a given transition set. The stability operator allows us to specify how and how far time has to unfold for the truth value of a sentence at a moment to become settled and thereby enables a perspicuous treatment of future contingents.

The transition semantics generalizes and extends both Peirceanism and Ockhamism. Both accounts are limiting cases of the transition approach that can be obtained by placing suitable restrictions on the transition sets employed in the semantic evaluation. Restricting the semantic evaluation to the empty transition set yields Peirceanism with its sole dependence on the moment parameter, while a restriction to maximal consistent transition sets yields Ockhamism with its dependence on moment-history pairs. On both accounts, stability collapses into truth. Operating on the whole range of consistent, downward closed transition sets provided by a BT structure, the transition semantics with its stability operator gains expressive means that are not available on either of those accounts and provides a fine-grained picture of the interrelation of modality and time.

## Footnotes

1 See Ploug and Øhrstrøm (2012) for the Kripke-Prior letters of 1958 and Øhrstrøm and Hasle (2011) for a broader overview.

2 So-called *Thin Red Line* approaches [as, for example, put forth in Øhrstrøm (2009)], which single out one of the possible futures as ‘the real future’, will be ignored here. For a critical discussion of the Thin Red Line, see Belnap et al. (2001), ch. 6.

3 From a philosophical perspective, it might seem favorable to assume that the truth values of the propositional variables depend only on the moment parameter, presupposing that atomic sentences do not contain traces of modality or futurity. In order to preserve the substitution property of the resulting logic, however, we have the truth values of the propositional variables in a BT model depend on all parameters of truth.

4 For Peirceanism and Ockhamism, see Prior (1967), pp. 122–134 and Thomason (1970), pp. 267–271.

5 Transitions as defined here are a special case of the more general notion of a transition provided in Belnap (2005), where a transition is defined as a pair $\langle I,O\rangle$ with $I,O \subseteq M$ fulfilling certain conditions. For the definition of the notion of a transition, see also Müller (2014).

6 Instead of restricting the definition of a transition $\langle m\!\rightarrowtail \!H\rangle$ to the case in which the initial *m* is a branching point, we could provide a more general definition of a transition by defining a transition for any arbitrary moment $m \in M$. The restriction we use is semantically motivated: if ${\varPi }_m = \{{\mathsf {H}}_m\}$, the transition $\langle m\!\rightarrowtail \!H\rangle$ does not exclude any history when it is part of a transition set, and we do not allow for a difference in truth value without change in the set of admitted histories as triggered by a branching point. If $\mathcal {M}=\langle M,<\rangle$ is a linear BT structure, which does not contain a branching point, the set $\mathsf {trans}(\mathcal {M})$ is the empty transition set, denoted by $\mathsf {\emptyset _{Tr}}$.

7 With the notion of the downward completion ${{\mathsf {d}}}{{\mathsf {c}}}(T)$ of a transition set *T* at our disposal, we can spell out in terms of transitions only what it means for a transition set to be consistent, without referring to the set ${\mathsf {H}}(T)$ of histories allowed by the transition set *T*. From Proposition 1 it immediately follows that a consistent set of transitions cannot contain two different transitions with the same initial since those would not be order related via $\prec$. When applied to the downward completion ${{\mathsf {d}}}{{\mathsf {c}}}(T)$ of a transition set *T*, that condition is even sufficient for the consistency of *T*. In other words, a set of transitions *T* is consistent if and only if its downward completion ${{\mathsf {d}}}{{\mathsf {c}}}(T)$ does not contain two transitions with the same initial. In the case of branching time, the two notions of consistency coincide, while they come apart in the case of branching space-time, as put forth in Belnap (1992), due to what is called modal correlations or ‘funny business’ (see Müller et al. (2008) and Müller (2014)).

8 As an anonymous referee has correctly pointed out, there are alternative ways to spell out the semantics without restricting the semantic evaluation to downward closed sets of transitions, treating instead all transition sets that allow exactly the same histories and hence specify the very same possible course of events as equivalent. Yet, generalizing the transition parameter is only possible at the cost of increasing the complexity of the semantic clauses and/or complicating reasoning about possible future extensions. In the absence of backward branching, closing transition sets downward seems to be the most natural choice.

9 In Sect. 4.1 we generalize the definition of a transition model by invoking the notion of a transition structure.

10 This is the semantic clause for the future operator provided in Müller (2014).

11 In addition to the strong future operator ${\mathsf {F}}$, which universally quantifies over future possibilities, we could also introduce a weak future operator ${\mathsf {f}}$ into our language, which is interpreted as an existential quantifier over future possibilities. For simplicity’s sake, we omit the operator ${\mathsf {f}}$ in the present paper.

12 The notion of a transition structure is the analogue of the definition of a bundled tree (see Definition 17): a transition structure comprises a set $ts$ of primitive transition sets, while a bundled tree comprises a set of primitive histories. In both cases, it is required that the respective ‘bundle’ spans the entire structure. As pointed out by an anonymous referee, rather than requiring every moment $m \in M$ to be compatible with some transition set in $ts$, we could alternatively require every history $h \in \mathsf {hist}(\mathcal {M})$ to be allowed by some such set, which would rule out incomplete Ockhamist transition structures with $ts \subsetneq \{{{\mathsf {T}}}{{\mathsf {r}}}(h)\in \mathsf {dcts}(\mathcal {M})\mid h \in \mathsf {hist}(\mathcal {M})\}$ from the outset (cf. Sect. 4.3). Our reason for preferring the more general definition over the stronger alternative is that BT structures are defined in terms of moments rather than in terms of histories. Histories are defined elements in a BT structure.

13 Consider a BT structure $\mathcal {M}$ that contains a history $h^*$ isomorphic to the natural numbers ${\mathbb {N}}$ such that at any moment $m_i \in h^*$ with $i \in {\mathbb {N}}$, there is a history $h_i$ such that $h^*\perp _{m_i} h_i$, and $\mathsf {hist}(\mathcal {M})= \{h^*\} \cup \{h_i\mid i \in {\mathbb {N}}\}$. The structure $\mathcal {M}^{ ts }$ with $ts = \{{{\mathsf {T}}}{{\mathsf {r}}}(h_i) \mid i \in {\mathbb {N}}\}$ is a transition structure according to Definition 13, but it is not an Ockhamist transition structure as $ts = \mathsf {mcts}(\mathcal {M})\setminus \{{{\mathsf {T}}}{{\mathsf {r}}}(h^*)\}$.

14 For a proof of that claim, see Burgess (1979) and Reynolds (2002). In Reynolds (2002), the sentence $\gamma := \mathsf {\Box _o}\mathsf {G_o}(p \rightarrow \mathsf {\Diamond _o}\mathsf {F_o}p) \rightarrow \mathsf {\Diamond _o}\mathsf {G_o}(p \rightarrow \mathsf {F_o}p)$ is provided, and it is shown that $\vDash _{{\mathsf {o}}}\gamma$ but . The problem is connected with the so-called limit closure property of complete bundled trees.

15 Note that the transition semantics also allows for consistent, downward closed transition sets that do not contain a greatest element.

16 For a discussion of the problem that a context of use fails to provide an initial value for the Ockhamist history parameter, see Belnap et al. (2001).

17 The helpful term ‘postsemantics’ was coined by MacFarlane (2003).

18 Belnaps’s theory of double time references makes use of a similar idea. See Belnap (2002).
